# Disentangling the effects of a century of eutrophication and climate warming on freshwater lake fish assemblages

**DOI:** 10.1371/journal.pone.0182667

**Published:** 2017-08-04

**Authors:** Peter C. Jacobson, Gretchen J. A. Hansen, Bethany J. Bethke, Timothy K. Cross

**Affiliations:** 1 Minnesota Department of Natural Resources, Park Rapids, Minnesota, United States of America; 2 Minnesota Department of Natural Resources, St. Paul, Minnesota, United States of America; 3 Minnesota Department of Natural Resources, Duluth, Minnesota, United States of America; 4 Minnesota Department of Natural Resources, Hutchinson, Minnesota, United States of America; University of Connecticut, UNITED STATES

## Abstract

Eutrophication and climate warming are profoundly affecting fish in many freshwater lakes. Understanding the specific effects of these stressors is critical for development of effective adaptation and remediation strategies for conserving fish populations in a changing environment. Ecological niche models that incorporated the individual effects of nutrient concentration and climate were developed for 25 species of fish sampled in standard gillnet surveys from 1,577 Minnesota lakes. Lake phosphorus concentrations and climates were hindcasted to a pre-disturbance period of 1896–1925 using existing land use models and historical temperature data. Then historical fish assemblages were reconstructed using the ecological niche models. Substantial changes were noted when reconstructed fish assemblages were compared to those from the contemporary period (1981–2010). Disentangling the sometimes opposing, sometimes compounding, effects of eutrophication and climate warming was critical for understanding changes in fish assemblages. Reconstructed abundances of eutrophication-tolerant, warmwater taxa increased in prairie lakes that experienced significant eutrophication and climate warming. Eutrophication-intolerant, warmwater taxa abundance increased in forest lakes where primarily climate warming was the stressor. Coolwater fish declined in abundance in both ecoregions. Large changes in modeled abundance occurred when the effects of both climate and eutrophication operated in the same direction for some species. Conversely, the effects of climate warming and eutrophication operated in opposing directions for other species and dampened net changes in abundance. Quantifying the specific effects of climate and eutrophication will allow water resource managers to better understand how lakes have changed and provide expectations for sustainable fish assemblages in the future.

## Introduction

Climate change and eutrophication are potent environmental stressors that are altering the fundamental ecological processes that structure freshwater communities throughout the world [1,2]. Significant shifts in thermal and nutrient regimes of lakes are manifested from phytoplankton and periphyton communities, up through zooplankton and benthic invertebrates, and ultimately to fish [3,4]. Since fish integrate the effects of stressors from lower trophic levels, they are valuable organisms for understanding the consequences of large-scale environmental changes [5]. Disentangling the effects of these multiple stressors is required to fully understand the consequences of climate warming and eutrophication on freshwater ecosystems [6,7] and the fish communities they support.

As climate changes the thermal structure of lakes, fish are influenced through a number of pathways and processes [8,9]. Epilimnetic warming affects shallow-dwelling taxa, while altered thermal and oxygen stratification regimes affect hypolimnetic dwellers [10,11]. Although effects of climate change on freshwater fish from all thermal guilds have been documented [8,12], there is still uncertainty about lake ecosystem responses and effects are likely not uniform across all lakes or across taxa [4,13]. Within geographic regions, coherence of lake temperature, solar radiation, and other similar variables can vary considerably [14]. Lake size, depth, and water clarity play a large role in how lakes respond to climate change [15–17]. Because lake size, depth, and water clarity play a large role in structuring fish communities [18], differences in these characteristics across lakes must be accounted for before the impacts of climate change can be isolated.

Eutrophication has substantially altered fish assemblages around the world [19] and can confound community responses to climate change even further [20,21]. Excessive nutrient loading can substantially change habitat-related properties that can determine fish assemblages. For example, increased phytoplankton-based turbidity shades out rooted macrophytes [22] that are critical habitat for phytophilic fish taxa. Increased periphyton can degrade spawning substrates [23] and reduce habitat for many benthophilic fish taxa. Increased primary productivity also reduces hypolimnetic oxygen concentrations for many fish [24]. Many of the eutrophication-related responses have the potential to mask or exacerbate fish assemblage responses to a changing climate [3].

Eutrophication and climate warming have affected many lakes in Minnesota [25,26]. Native prairies in the southern and western portion of the state were converted to row-crop agriculture in the late 1800s and early 1900s [27]. Nutrient and sediment loadings into lakes increased significantly as agricultural practices (e.g. enhanced drainage, increased fertilization, and concentrated livestock production) intensified in the 1950s [28]. By the late 1900s, many lakes in the agricultural portion of Minnesota were severely impaired by eutrophication [29]. Lakes in the northern portion of the state have largely been spared from nutrient enrichment because their watersheds have largely remained forested [25,30]. In addition, the climate has warmed across the entire state in recent years [31]. These extensive changes in climate and land use provide a natural experiment to quantify the effects of climate and eutrophication on fish in lakes.

Ecological niche modeling that incorporates specific effects of individual stressors has proven useful for disentangling multiple-stressor impacts on biotic communities [32,33]. Each stressor can enter the model if a quantifiable, representative variable can be identified. Individual taxa are modeled separately, which is useful since effects are stressor- and taxa-specific. Ecological niche models that include stressor variables generally describe realized Grinnellian niches [34] focused on existing occurrences or densities of animals in the environment, rather than fundamental niches based on laboratory-measured physiologic responses [35].

Understanding the specific effects of eutrophication and climate warming is of critical importance for water resource managers and policy makers. Minnesota’s lake fish taxa differ in their thermal and eutrophication tolerances [24,29] and responses to the combined influence of thermal change and eutrophication may not be straightforward. For example, the combined response of some taxa to the two stressors may offset each other when they act in opposite directions. Taxa-specific approaches that quantify the influence of multiple stressors concurrently are required for fully understanding the response of fish assemblages to environmental change. Here, taxa-specific ecological niche models were developed to quantify the influence of air temperature and nutrient concentrations on the contemporary abundance of lake fishes spanning a range of thermal and eutrophication tolerances. Then, lake productivities and climates were hindcast to a pre-disturbance period using existing land use models and historical air temperature data. Finally, historical fish assemblages in pre-disturbance conditions were reconstructed to estimate changes in fish species abundance and community composition over the previous century.

## Materials and methods

### Contemporary fish abundance estimation

Empirical ecological niche models were developed for 25 species of fish commonly captured in Minnesota Department of Natural Resources (MDNR) lake netting assessments (Table 1). The Minnesota Department of Natural Resources has the authority to regulate, manage, and undertake the scientific collection of fish in the waters of the state. Collection of fish for this study was completed under that authority and all guidelines and standard procedures for field sampling methods were followed, including the release of live fish back into the lake. Lakes are considered public lands in the State of Minnesota. Relative abundance (catch per net, CPE) was determined from standardized MDNR gillnetting assessments on 1,577 lakes from 1993 through 2010. Gillnets, consisting of 75m of graded-mesh multifilament material (15m panels of 19, 25, 38, 51, and 64mm bar-measure mesh x 2m deep), were set overnight during the months of June, July, and August. The nets (n = 2 to 15 for each assessment based on lake size) were set near the lake bottom at or above the thermocline, generally targeting epilimnetic dwellers (primarily cool and warmwater taxa). Coldwater fish species are poorly sampled in these sets because they occur deeper in the hypolimnia and were not included in this analysis. Mean CPE of cool and warmwater species that were captured in at least 2% of lakes (species listed in Table 1) were calculated for individual lakes for the period from 1993 through 2010 (lakes were typically resampled every 5–10 years).

**Table 1:** Species list.

Species	Common Name	Guild
*Ambloplites rupestris*	rock bass	Intolerant Coolwater
*Ameiurus nebulosus*	brown bullhead	Intolerant Coolwater
*Esox lucius*	northern pike	Intolerant Coolwater
*Esox masquinongy*	muskellunge	Intolerant Coolwater
*Micropterus dolomieu*	smallmouth bass	Intolerant Coolwater
*Amia calva*	bowfin	Tolerant Coolwater
*Catostomus commersoni*	white sucker	Tolerant Coolwater
*Moxostoma anisurum*	silver redhorse	Tolerant Coolwater
*Moxostoma macrolepidotum*	shorthead redhorse	Tolerant Coolwater
*Perca flavescens*	yellow perch	Tolerant Coolwater
*Sander vitreus*	walleye	Tolerant Coolwater
*Ameiurus natalis*	yellow bullhead	Intolerant Warmwater
*Lepomis cyanellus*	green sunfish	Intolerant Warmwater
*Lepomis gibbosus*	pumpkinseed	Intolerant Warmwater
*Lepomis machrochirus*	bluegill	Intolerant Warmwater
*Micropterus salmoides*	largemouth bass	Intolerant Warmwater
*Ameiurus melas*	black bullhead	Tolerant Warmwater
*Aplodinotus grunniens*	freshwater drum	Tolerant Warmwater
*Cyprinus carpio*	common carp	Tolerant Warmwater
*Ictalurus punctatus*	channel catfish	Tolerant Warmwater
*Ictiobus cyprinellus*	bigmouth buffalo	Tolerant Warmwater
*Morone chrysops*	white bass	Tolerant Warmwater
*Notemigonus crysoleucas*	golden shiner	Tolerant Warmwater
*Pomoxis annularis*	white crappie	Tolerant Warmwater
*Pomoxis nigromaculatus*	black crappie	Tolerant Warmwater

List of fish species used for ecological niche modeling, with eutrophication tolerance and thermal guild assigned by ecological niche modeling results from this study.

### Ecological niche modeling

Taxa-specific ecological niche models were developed using generalized additive models (GAMs). The effects of several environmental and ecological stressor variables on the relative abundance of each species were quantified with the models. GAMs are particularly useful for modeling the ecological niche of biological organisms because they directly incorporate nonlinear smoothing to describe the effects of predictors on the response variable [36] and are increasingly being used to model aquatic communities [37,38]. Two proxy variables that represented the ecological stressors of climate warming and eutrophication along with three environmental variables (lake size, depth, and alkalinity) known to affect fish populations in lakes [39,40] were used as predictor variables in the models. Taxa-specific model responses were used to develop tolerance classifications for climate and eutrophication by examining response shapes and peaks in relative abundance [41], along with considering previous classifications of cool and warmwater fishes [10] and eutrophication [29]. Taxa were grouped into two thermal preference classes (coolwater and warmwater) and two classes based on their tolerance to eutrophication (intolerant and tolerant).

Mean annual air temperature (MAT), which integrates a number of ecological effects across all seasons, was used as a proxy variable that captured the effects of climate on fish assemblages. Lake-specific air temperature data were developed from high resolution spatial interpolations of historic air temperatures available from the Oregon State University PRISM Climate Group (http://prism.oregonstate.edu, retrieved 3 Dec 2015). Air temperatures were estimated for the geographical center of each lake for the period from 1981–2010 using the geoknife package in R [42] and mean values over the entire 30 year period were calculated for each lake.

Phosphorus was selected as a proxy variable that captured the effects of eutrophication because it is the primary limiting nutrient in many lakes, including Minnesota [29,30]. Mean summer epilimnetic total phosphorus concentration (TP) and total alkalinity field measurements were obtained from Minnesota Pollution Control Agency (MPCA) and MDNR databases for the period from 1993 through 2005. Epilimnetic water samples were collected using either a 2 m long, 32 mm diameter polyvinyl chloride (PVC) pipe integrated sampler or surface grab samples. Chemical analyses of total P were performed by the Minnesota Department of Agriculture, Minnesota Department of Health, or at MPCA-approved laboratories. MPCA data is available online (https://www.pca.state.mn.us/water/water-quality-data, accessed 23 Jun 2017).

The *mgcv* (multiple generalized cross validation package [43] in the statistical program R Version 3.2.4 (R Core Team 2016) was used for the development of the GAMs. The *mgcv* implementation fitted a series of penalized regression splines as smoothing functions for each predictor variable and suggested degrees of freedom for smooth terms by minimizing the generalized cross validation score. Fish relative abundances were transformed as log_e_(CPE+0.1) and by z-scores (subtracting the species-specific mean and dividing by the species-specific standard deviation) to allow for standardized comparisons among species. Lake size (ha) and depth (m) were log transformed and total alkalinity was square root transformed to normalize their distributions. Response surfaces of relative abundances were estimated from joint smooths of MAT and TP. The joint smooths allowed for the visualization of how responses of each variable changed as a function of the other variable. Individual responses can be visualized as slices through the response surface at specific values of the other variable. These interaction of MAT and TP can also be visualized as the shape of the slices change as a function of the other variable. Environmental variables (lake size, depth, and total alkalinity) were entered into the model as single variable smooth terms. A Gaussian family, with an identity link function, was used for each GAM and a dimension basis of k = 4, was used to limit the degrees of freedom for each smooth term [43].

### Pre-disturbance condition and fish assemblage reconstruction

Pre-disturbance lake conditions were estimated for climate and eutrophication variables which provided inputs into species-specific ecological niche models. Pre-disturbance climate was calculated from historical air temperatures retrieved from the PRISM data set using identical methods as the contemporary climate data for comparison to present-day conditions. Annual lake-specific air temperatures from 1896–1925 (a thirty year period of the same duration as the contemporary period) were processed using the geoknife package in R [42], and mean values over the entire 30 year period were calculated for each lake.

Pre-disturbance land use corresponded to the late 1800s when prairies were first converted to agriculture and urban areas started to develop in Minnesota. Pre-disturbance eutrophication status was estimated by hindcasting the lake phosphorus model developed by Cross and Jacobson [30] to undisturbed conditions for each lake. The model directly uses a watershed land use disturbance variable that can be set to zero to characterize undisturbed conditions. First, a linear version was derived from the original, nonlinear model presented in Fig 2 of Cross and Jacobson [30]:

$$TP=e^{4.05-0.454\;\ln\left(depth\right)-0.472outwash+1.60disturbance}$$ (1)

where *TP* = mean summer epilimnetic total phosphorus concentration (μg/l), *depth* = maximum lake depth (m), *outwash* = proportion of glacial outwash soils in watershed, *disturbance* = proportion of disturbed land uses (agricultural, urban, and mining) in watershed. Assuming depth and proportion of outwash soils remained constant over both periods, estimation of pre-disturbance lake productivity collapses to:

$${TP}_{p}=e^{\ln\left(TPc\right)-1.60disturbance}$$ (2)

where *TP*_*p*_ = pre-disturbance mean summer epilimnetic phosphorus concentration (μg/l), *TP*_*c*_ = contemporary, observed mean summer epilimnetic phosphorus concentration (μg/l), and *disturbance* = proportion disturbed land uses (agricultural, urban, and mining) currently in watershed. Watershed delineations were available for 1,236 lakes in the analysis. Contemporary values of land uses were calculated with the 2001 National Land Cover Database [44]. Level 1 continental ecoregions [45] were also used to group lakes into the three major land types within the state (Northern Forests, Eastern Temperate Forests, and Great Plains). Level 1 ecoregions names were simplified from Northern Forests to forest, Eastern Temperate Forests to transition, and Great Plains to prairie ecoregions.

Pre-disturbance fish assemblages were estimated by using the derived historical conditions as inputs to taxa-specific ecological niche models. Lake-specific CPEs were calculated from the fitted GAM models for each species to explore changes in abundance from pre-disturbance to contemporary conditions. The effects of climate warming and eutrophication on the relative abundance of each taxa were examined independently by using pre-disturbance conditions for one stressor while holding the other at contemporary levels, and then collectively by using pre-disturbance levels for both stressors.

## Results

### Ecological niche models

Generalized additive models described between 1.2 and 57.6% of deviance in fish relative abundance for the 25 species (S1 Table). Joint fits of the effects of mean annual temperature and total phosphorus concentration on relative abundance were statistically significant (α≤0.05) for all 25 taxa (S1 Table). Taxa-specific combinations of lake size, maximum depth, and total alkalinity were also significant variables in many of the models (S1 Fig and S1 Table).

**Fig 1:**
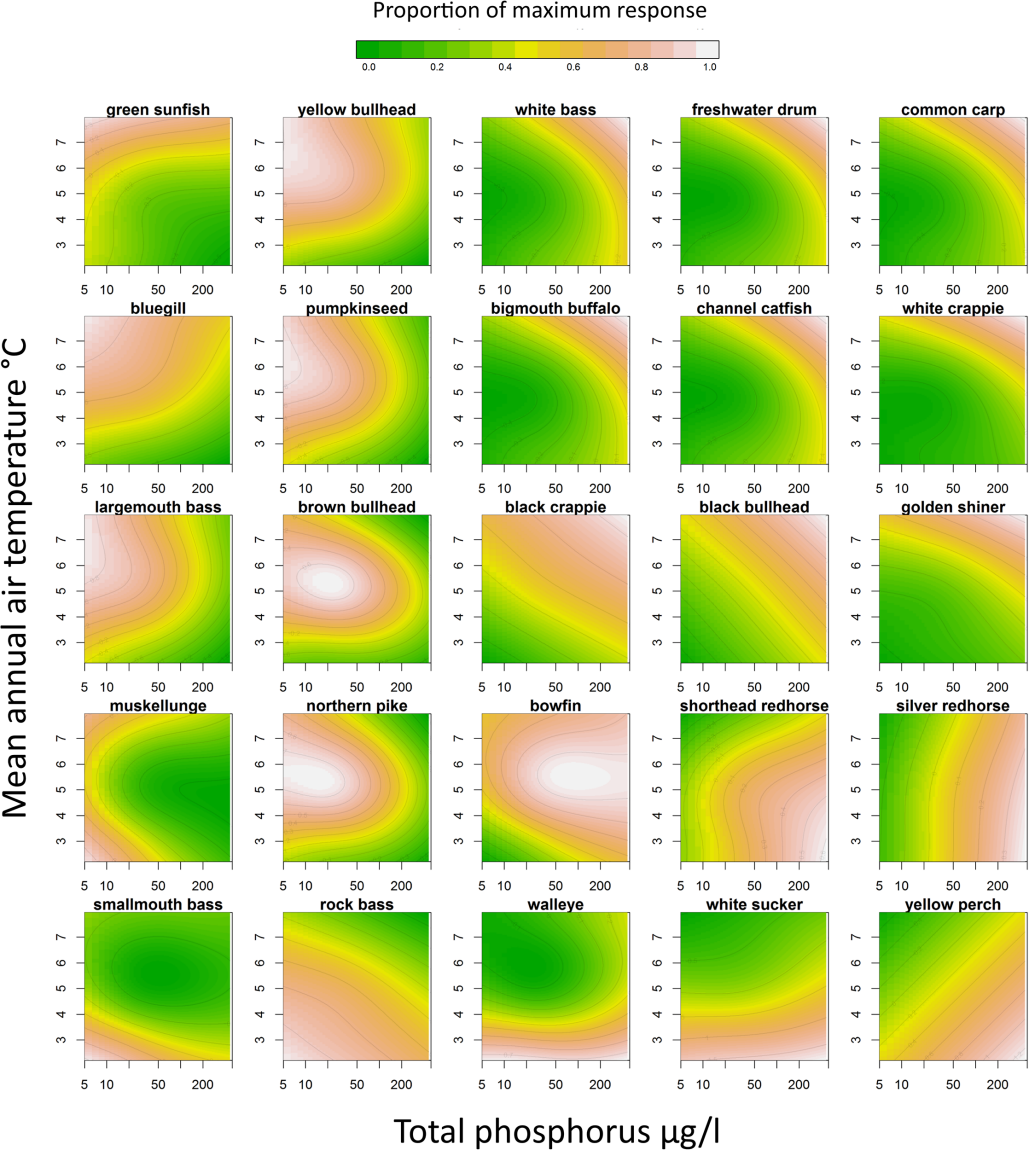
Niche model response surfaces. Generalized additive model response surfaces of the joint effects of mean annual temperature (°C) and mean summer epilimnetic total phosphorus concentrations (μg/l) on the relative abundance of 25 fish species sampled in 1,577 Minnesota lakes. Relative abundance was standardized (z-score of log_e_(CPE+0.1)) and rescaled by proportion of maximum fitted response.

Response shapes of the joint effects of climate and productivity varied considerably between species (Fig 1). Some had readily apparent domes of peak relative abundance responses (yellow bullhead, pumpkinseed, largemouth bass, brown bullhead, northern pike, and bowfin), which indicated that the range of climate and lake productivities in Minnesota encompassed a prime portion of their niche space. The peak abundance of other taxa occurred on the edge of the climate and productivity niche space (e.g. green sunfish, common carp, smallmouth bass and yellow perch), which indicated that some of their optimal niche conditions extend beyond the range of temperatures and productivities observed in Minnesota. Species with peak relative abundance responses at mean annual temperatures greater than 6°C were considered to be warmwater fishes and included a number of species in the Centrarchidae (sunfish and basses) and Ictaluridae (catfishes) families (Table 1). The group of species with peak relative abundance responses of mean annual temperatures less than 6°C were considered to be coolwater fishes and included a number of Esocidae (pikes) and Percidae (perch and walleyes) (Table 1). Tolerance to eutrophication was assigned based on the location of abundance response peaks relative to the total phosphorus axis. Species most abundant in high productivity lakes (>25μg/l total phosphorus) were classified as tolerant (Table 1) and included common species such as walleye, yellow perch, white sucker, black bullhead, and common carp. Intolerant taxa (most abundant in low productivity lakes less than 25μg/l total phosphorus) included common species such as northern pike, rock bass, muskellunge, smallmouth bass, yellow bullhead, bluegill, and largemouth bass.

### Changes in air temperatures and lake productivities

**Fig 2:**
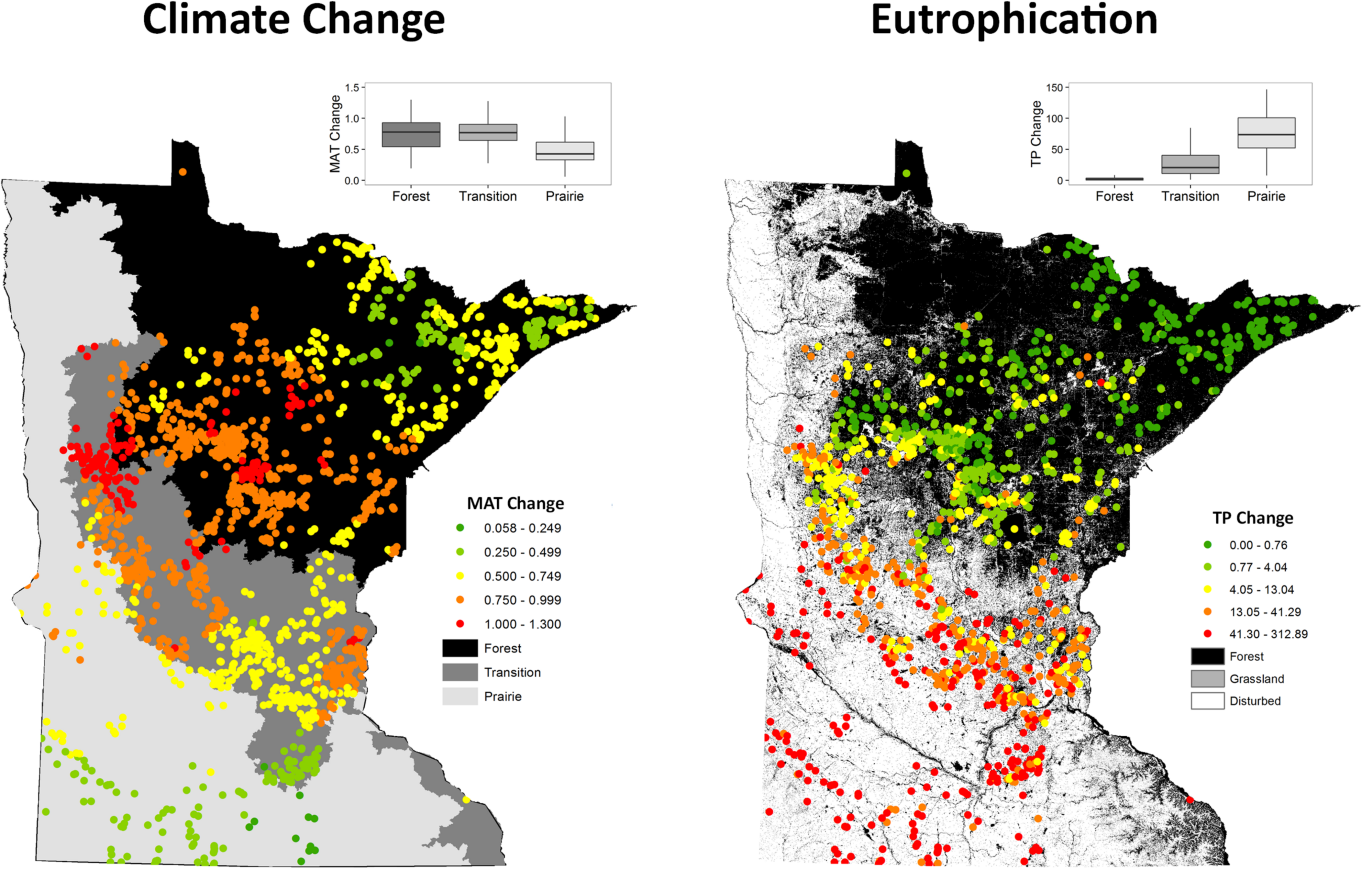
Climate and lake productivity changes. Changes in Minnesota lakes from (a) climate warming (MAT—mean annual air temperature °C) and (b) eutrophication (TP—mean summer epilimnetic total phosphorus concentrations μg/l) from pre-disturbance (1896–1925) to contemporary periods (1981–2010) for 1,236 lakes in Minnesota. Figure insets are box plots of interquartile ranges. The background in (a) represents Level 1 ecoregions [45] and (b) simplified land cover classes derived from the 2001 National Land Cover Database [44].

Mean annual air temperatures increased from pre-disturbance (1896–1925) to contemporary (1981–2010) times, although the magnitude of increase varied substantially between lakes and ecoregions (Fig 2). Temperature increases were greatest for the north-central portion of the state, where the mean annual air temperature for some lakes warmed by more than 1°C. Lakes in southwestern Minnesota experienced the least warming, with increases in MAT generally less than 0.5°C. Consequently, lakes in the forested and transition ecoregions experienced the greatest warming (mean increases of 0.75°C and 0.77°C in MAT, respectively) and lakes in the prairie ecoregion experienced less (mean increase of 0.48°C).

Hindcasted total phosphorus concentrations indicated that lakes in the transition and prairie ecoregions have become more productive (Fig 2). Those ecoregions experienced the greatest land use change with the majority of native prairies and transition forests converted to agriculture and urban areas (as represented by disturbed land uses in the background of Fig 2). Although lakes in the prairie ecoregion were inherently more productive before land use changes, many have prominently higher contemporary total phosphorus concentrations and large magnitudes of change (mean TP increase of 97μg/l). Losses of forest and grasslands also resulted in substantially higher lake productivities in the transition ecoregion (mean TP increase of 40μg/l). Lakes in lands that are currently forested have experienced little change in productivity and total phosphorus concentrations have remained low (mean TP increase of 3μg/).

**Fig 3:**
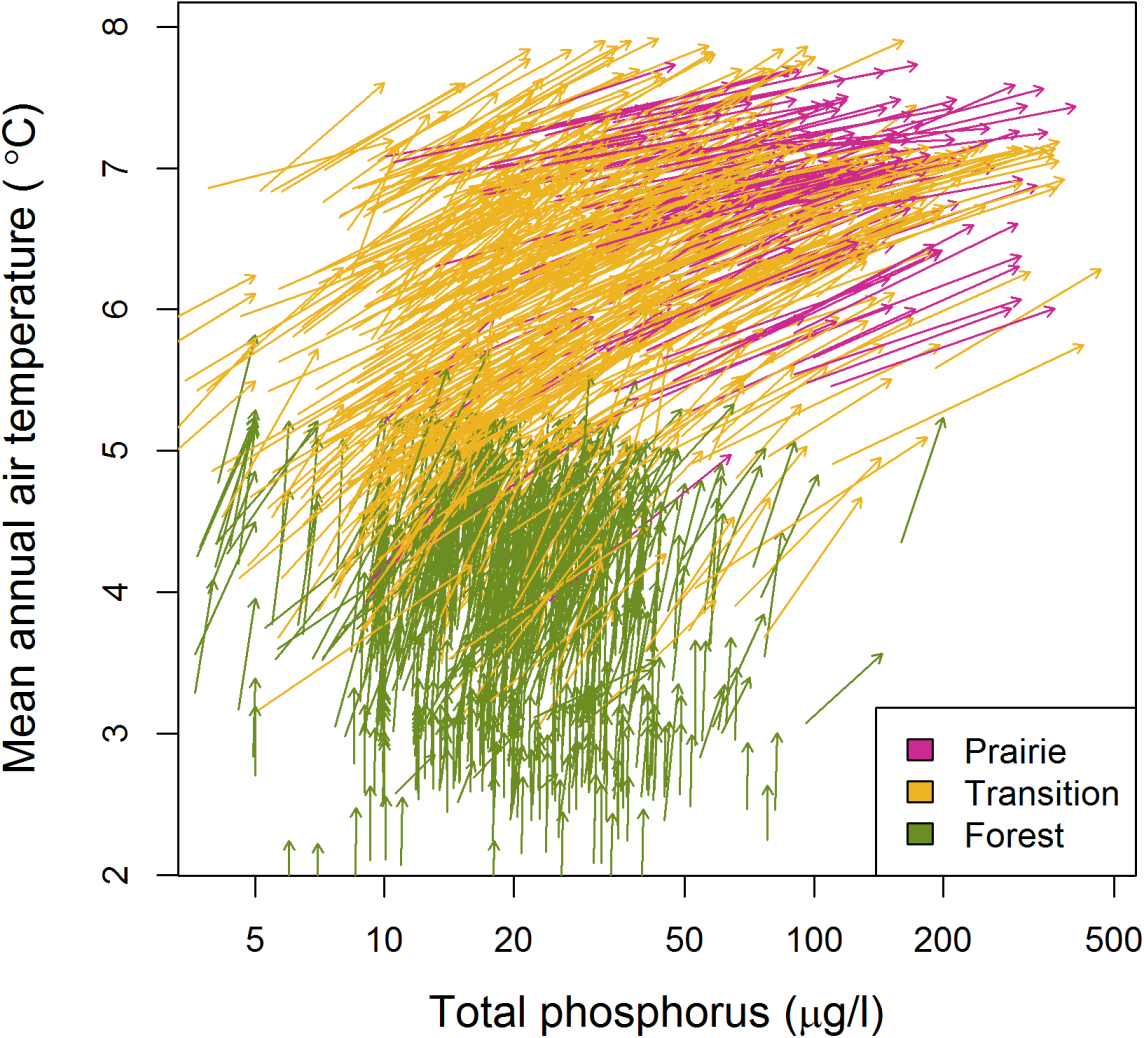
Niche space changes. Trajectories of estimated 2-dimensional niche space changes in climate (mean annual air temperature °C) and productivity (mean summer epilimnetic total phosphorus concentration μg/l) in 1,236 Minnesota lakes from 1896 to 2010.

Trajectories of individual lakes within a 2-dimensional climate and productivity niche space (Fig 3) illustrated how Minnesota lakes have changed markedly in the past century. Vectors of climate warming moved all lakes upward into warmer portions of the niche space. In addition, many lakes in the prairie and transition ecoregions moved substantially to the right into portions of the niche space with higher productivities. Net-result diagonal trajectories indicated that many lakes in the transition and prairie ecoregions moved substantially across the niche space along both climate and productivity axes. Lake-specific effects of climate and eutrophication can be visualized as a vector that traverses across the same 2-dimensional niche space of response surfaces for each taxa presented in Fig 1.

### Changes in fish assemblages

**Fig 4:**
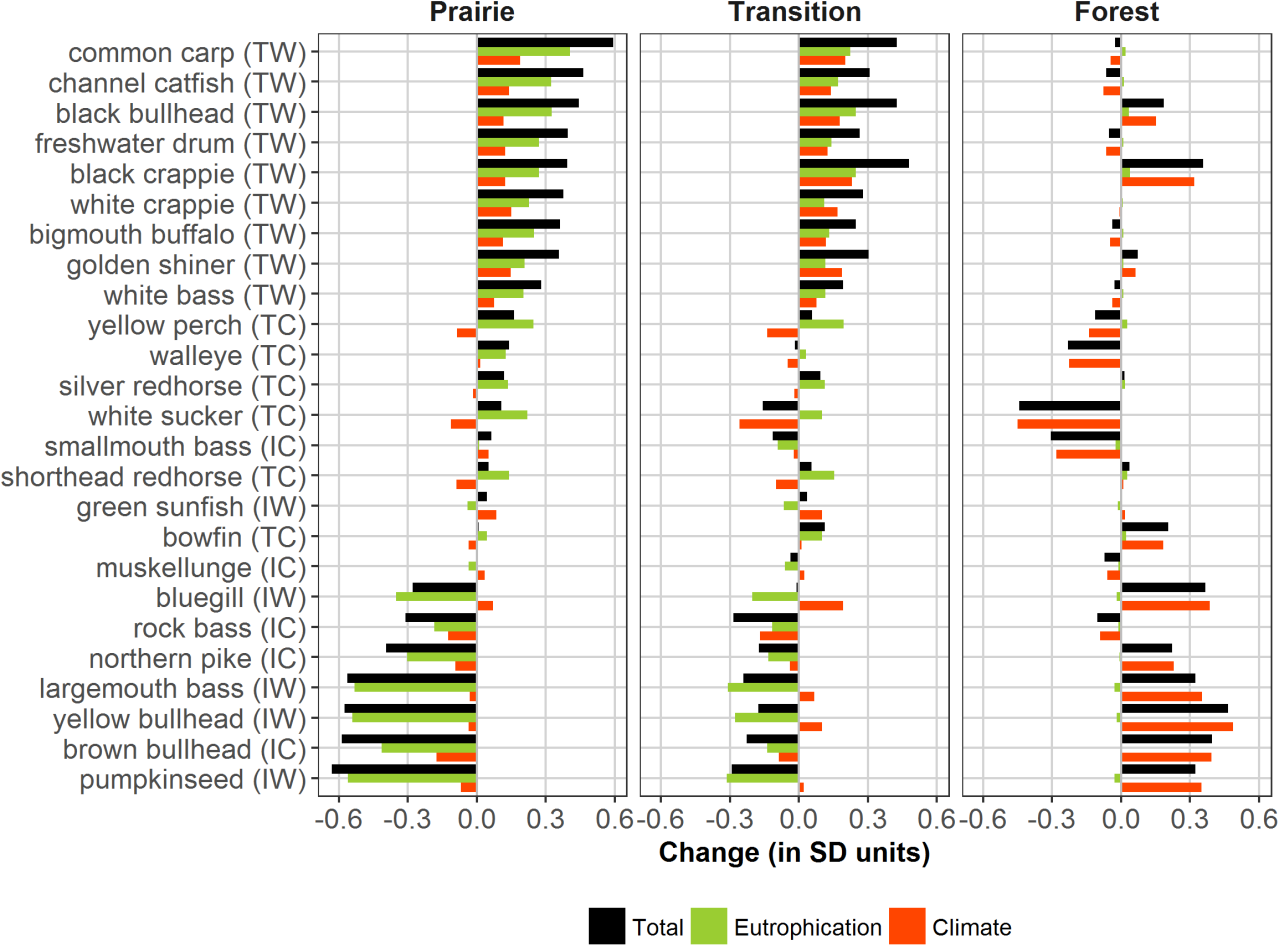
Stressor-specific changes in abundance. Stressor-specific changes in model-estimated standardized relative abundance (z-score of log_e_(CPE+0.1)) presented in units of standard deviations for 130 Minnesota lakes in the prairie, 508 lakes in the transition, and 598 lakes in the forest ecoregions from 1896 to 2010. TW = eutrophication-tolerant warmwater, TC = eutrophication-tolerant coolwater, IW = eutrophication-intolerant warmwater, and IC = eutrophication-intolerant coolwater.

Stressor-specific changes in modeled fish relative abundance varied considerably by species and ecoregion (Fig 4). The direction and magnitude of change also varied by stressor. Taxa experiencing the largest changes in abundance were those for which the effects of both temperature and phosphorus operated in the same direction. Conversely, the effects of temperature and phosphorus operated in opposing directions for some taxa and dampened net changes in abundance. Species compositions within each of these classes of responses varied substantially across ecoregions.

For most taxa, hindcast changes in abundance were largest in prairie lakes, where both climate and productivity shifts were substantial. All nine eutrophication-tolerant warmwater species were estimated to have increased in abundance from increased phosphorus and temperature, while six intolerant species (rock bass, northern pike, largemouth bass, yellow bullhead, brown bullhead, and pumpkinseed) declined over the period. Stressors operated in opposite directions for a total of eight species, several of which experienced little change in abundance. Eutrophication-tolerant coolwater yellow perch benefited from increased total phosphorus, but warmer temperatures were detrimental. Conversely, intolerant warmwater bluegill benefited from warming temperatures, but gains in abundance were overwhelmed by detrimental effects of increased phosphorus. In general, eutrophication produced larger effects on relative abundance than climate warming for most taxa in the prairie ecoregion.

Fish assemblages in the transition ecoregion also experienced significant changes after disturbance. Taxa-specific responses were similar to the prairie ecoregion with eutrophication-tolerant warmwater species benefitting most from increased temperatures and phosphorus concentrations. Increased phosphorus concentrations were detrimental to a number of eutrophication-intolerant species and increased temperatures were beneficial to warmwater taxa. Tolerant coolwater yellow perch also benefited from increased lake productivities, but increased temperatures were detrimental. Intolerant warmwater bluegill gains in abundance from increased temperatures were completely offset by losses from increased phosphorus concentrations and resulted in no net change. A total of 12 species in the transition ecoregion were affected by stressors that operated in opposite directions.

Fishes in the forested ecoregion experienced changes primarily driven by temperature increases in the past century. Warmwater fishes such as black crappie, bluegill, largemouth bass, pumpkinseed, and yellow bullhead increased in abundance. The greatest abundance declines were seen within coolwater species such as white sucker, walleye, yellow perch, and smallmouth bass. The number of species affected by stressors that operated in opposite directions was large (15), but the net effect of increased phosphorus was very small in all cases.

Grouping of taxa by thermal preference and eutrophication tolerance summarized fish assemblage changes that have occurred in the past century (Table 2). Warmwater taxa increased in all three ecoregions. In the prairie ecoregion, increased temperatures and phosphorus shifted many intolerant cool- and warmwater fishes to tolerant warmwater assemblages. Similar shifts occurred in the transition ecoregion. Lakes in the forest ecoregion of Minnesota experienced shifts almost exclusively due to warming temperatures, with increased abundance of both intolerant and tolerant warmwater taxa.

**Table 2:** Changes in abundance by tolerance and guild.

	Forest		Transition		Prairie	
Tolerance/Guild	Pre	Post	Pre	Post	Pre	Post
Intolerant Coolwater	27.1	26.0	22.3	17.6	16.0	10.4
Tolerant Coolwater	31.3	26.6	22.0	20.8	21.5	20.7
Intolerant Warmwater	15.6	21.0	27.0	22.1	19.5	11.6
Tolerant Warmwater	26.0	26.3	28.7	39.5	42.9	57.2

Mean relative abundance of fish as percent composition of eutrophication tolerance and thermal guild by ecoregion for pre-disturbance reconstructed (1896–1925) and post-disturbance contemporary (1981–2010) periods for 130 lakes in the prairie, 508 lakes in the transition, and 598 lakes in the forest ecoregions in Minnesota.

## Discussion

A century of eutrophication and climate warming profoundly affected lakes in Minnesota and consequently, their fish assemblages. Conversions of native prairies and forests to agricultural and urban uses increased nutrient concentrations in the prairie and transition ecoregions of the state. In response, abundances of eutrophication-tolerant fish likely increased. Climate also warmed in the past century throughout the entire state and reconstructed warmwater fish abundances increased accordingly. Trajectories of lake-specific changes spanned an extraordinary range of the 2-dimensional niche space represented by these two stressors in Minnesota. Together, eutrophication and climate warming likely drove an expansion of tolerant warmwater fish abundance at the expense of intolerant coolwater taxa. Conversely, climate and productivity shifts produced opposing effects on abundance of several species. For example, tolerant coolwater yellow perch likely benefitted from lake productivity increases, but climate warming was detrimental. Intolerant warmwater bluegill benefitted from climate warming, but gains in abundance were overwhelmed by negative effects of eutrophication. Disentangling the sometimes opposing, sometimes compounding, effects of these two important ecological stressors was critical for understanding changes in fish assemblages. Taxa-specific ecological niche models and explicit identification of stressor-specific tolerance guilds [46] were valuable for disentangling these multiple stressors.

Eutrophication drove changes in modeled lake fish assemblages in the prairie and transition ecoregions of Minnesota more than climate. Vegetated fish habitat in the many shallow lakes of the prairie was likely reduced by nutrient enrichment [22]. Shifts from stable, clear-water vegetated states to unstable, turbid states are typical responses of shallow lakes to eutrophication [47]. Dense phytoplankton blooms in nutrient-enriched lakes shade out stands of rooted macrophytes, critical habitat for phytophilic taxa such as bluegill, largemouth bass, and northern pike. Conditions that favored eutrophication-tolerant warmwater common carp and black bullheads likely exacerbated the effects of eutrophication by the destructive nature of their benthivorous feeding behaviors on rooted plants [48,49]. The relatively greater influence of land use change over that of climate was consistent with other studies of freshwater systems [7,50]. Although the effects of climate warming on fish in the prairie ecoregion was less strong, when added to the effects of eutrophication the combination of both stressors likely exerted substantial change on fish communities in prairie lakes.

Climate drove modeled fish assemblage change in forested ecoregion lakes more than eutrophication. Watersheds of these lakes remained largely undisturbed and forested [30] resulting in minimal eutrophication. A number of climate-driven processes likely shaped changes in fish abundance in the forest ecoregion including increased duration of lake stratification, reduction in duration of ice cover, and lengthening of the growing season [4]. Epilimnetic thermal habitat for warmwater fish likely improved and increased abundance of centrarchid species has been documented in Minnesota [51], Wisconsin [40], and Ontario [52] lakes. Reduced duration of ice cover from shortened winters possibly affected recruitment of some species, as in the case of reduced reproductive success of yellow perch in Lake Erie following warm winters [53]. Additionally, advancement in spring phenology may have changed the timing of plankton and zooplankton production and availability for larval fish, resulting in predator-prey mismatch issues which may disturb recruitment of fishes [54].

Although sequence and timing of individual stressor effects was not determined in this study, evidence exists that eutrophication was most important during the first part of the century and climate became more significant in recent years. Increases in mean annual temperatures in the state have accelerated since 1980 [31]. Many lakes in the agricultural portion of Minnesota were already severely impaired from eutrophication by that time [29]. Recent trends in Minnesota fish abundances [51] were consistent with the climate component of the changes estimated in this study. Statewide increased abundances since 1970 were noted for warmwater taxa such as bluegill, black crappie, and largemouth bass and decreased abundances for coolwater yellow perch and white sucker. Statewide abundance trends observed for other species such as walleye, smallmouth bass, and northern pike were less consistent, but ecoregion-specific changes make comparisons with statewide trends difficult.

The effects of climate warming on fish are expected to continue in all three ecoregions. Lakes in the forested ecoregion are likely to maintain suitable thermal habitat for cool- and warmwater species even under extreme warming scenarios [40,55]. Thus, the effects of continued climate warming on cool- and warmwater fish species are likely to remain complex [56,57]. Hydrologic changes linked to climate change will affect lakes as rain and snow are delivered in less frequent but more severe events [31]. These hydrologic alterations affect runoff and nutrient loading, which are important factors determining TP concentrations in lakes. The confounding effects of climate on eutrophication will continue to be an important issue in the future [13,58].

Although ecological niche modeling was useful for disentangling the effects of climate and eutrophication, significant uncertainty remains for predicting fish assemblage responses. Model errors propagated through the phosphorus hindcasting model and fish assemblage reconstruction were likely present. Modeled niches were assumed to remain unchanged from historic to contemporary conditions [59]. Predictions based on environmental conditions that fall outside the bounds of measured observations is of concern as well [60,61]. In this study, the hindcasted estimates of temperature and lake productivity fell within the range of observed, contemporary conditions. Although correlative models projected onto other time periods or environments must always be treated with caution, such models represent an important approach available for prediction on a scale and resolution relevant to management decisions [62,63]. In addition, it is important to view lake community dynamics not only in the context of environmental variables, but also biotic interactions [64]. Considering species range shifts within a “bioclimate envelope” which encompasses both biotic interactions and climate have been suggested [60]. Biotic interactions were a significant limiting factor for a terrestrial invertebrate species in Europe, even to a greater extent than climatic variables [65]. In contrast, predicted extirpation of cisco populations in Wisconsin was found to be more driven by climate change than by invasions of rainbow smelt, an invasive competitor [66]. Community ecologists have also called for a greater link between environmental niche models and biological community interactions [67]. Incorporation of biotic interactions and accounting for changing niches could further explain unaccounted variation in the ecological niche models and offer great opportunities for future research.

### Management implications

Understanding the specific effects of climate and land use change on lakes and fish assemblages is critical for developing management and adaptation strategies for effective conservation [68,69]. For example, protecting resilient systems such as lakes with good water quality is a strategy that will ameliorate the effects of climate change on eutrophication-intolerant taxa [24]. Many lakes in the northern, forested portion of the state will likely remain sufficiently cool to support coolwater fish [55]. If water quality can be protected in those systems, populations of high-valued intolerant coolwater species such as muskellunge, northern pike, and smallmouth bass can be sustained. Implementing watershed protection strategies would be a specific climate adaptation strategy for those systems. The analysis also provides a framework for fisheries managers to develop species assemblage goals for lakes that are changing. For example, in prairie lakes with intensively farmed watersheds where restoration of water quality continues to be challenging [28], managing for eutrophication-tolerant warmwater taxa will likely be the most successful approach. However, in transition ecoregion lakes, managing for eutrophication-intolerant taxa could still be possible if water quality remediation efforts prove successful in less disturbed watersheds. The modeling will also allow lake and fisheries managers to demonstrate to the public on how lakes and fish assemblages have changed in the past century. Public understanding of the importance of both climate and land use change in structuring fish assemblages in lakes could provide the basis for enlightened support of effective water quality protection and climate adaptation efforts. Can management agencies accept the demise of taxa from stressors that are beyond their direct control such as climate warming, and concentrate on taxa that benefit from climate warming or benefit from the reversal of eutrophication through watershed restoration? Only when stressor-specific effects are disentangled will tailored management actions be possible and appropriate remediation and adaption efforts targeted effectively.

## Supporting information

S1 Table Taxa-specific model significance. P-values and percent deviance explained for generalized additive models of the relative abundance of 25 fish species predicted by mean annual temperature (MAT) and mean summer epilimnetic total phosphorus concentrations (TP), depth, area, and alkalinity sampled in 1,577 Minnesota lakes.
(XLSX)

S2 Table Model variable values. Lake specific data used for ecological niche models developed from 1,577 Minnesota lakes. Metadata describing each data field are described in the first portion of the Table.
(XLSX)

S1 Fig Taxa-specific model responses. Generalized additive model responses of mean annual temperature (MAT °C) and mean summer epilimnetic total phosphorus concentrations (TP μg/l), depth (m), area (ha), and alkalinity on the relative abundance of 25 fish species sampled in 1,577 Minnesota lakes. Species codes are defined in S1 Table and effective degrees of freedom for each smoothed fit are presented in the y-axis caption. Red lines represent lower 95% confidence interval bounds and green lines represent upper 95% confidence interval bounds.
(PDF)
