# Supplementary material for: Disentangling the effects of a century of eutrophication and climate warming on freshwater lake fish assemblages
Source: PLoS One. 2017 Aug 4;12(8):e0182667. doi: 10.1371/journal.pone.0182667 (PMC5544199; doi:10.1371/journal.pone.0182667)
Supplement: S1 Fig — Generalized additive model responses of mean annual temperature (MAT °C) and mean summer epilimnetic total phosphorus concentrations (TP μg/l), depth (m), area (ha), and alkalinity on the relative abundance of 25 fish species sampled in 1,577 Minnesota lakes. Species codes are defined in S1 Table and effective degrees of freedom for each smoothed fit are presented in the y-axis caption. Red lines represent lower 95% confidence interval bounds and green lines represent upper 95% confidence interval bounds. (PDF) [file pone.0182667.s003.pdf]

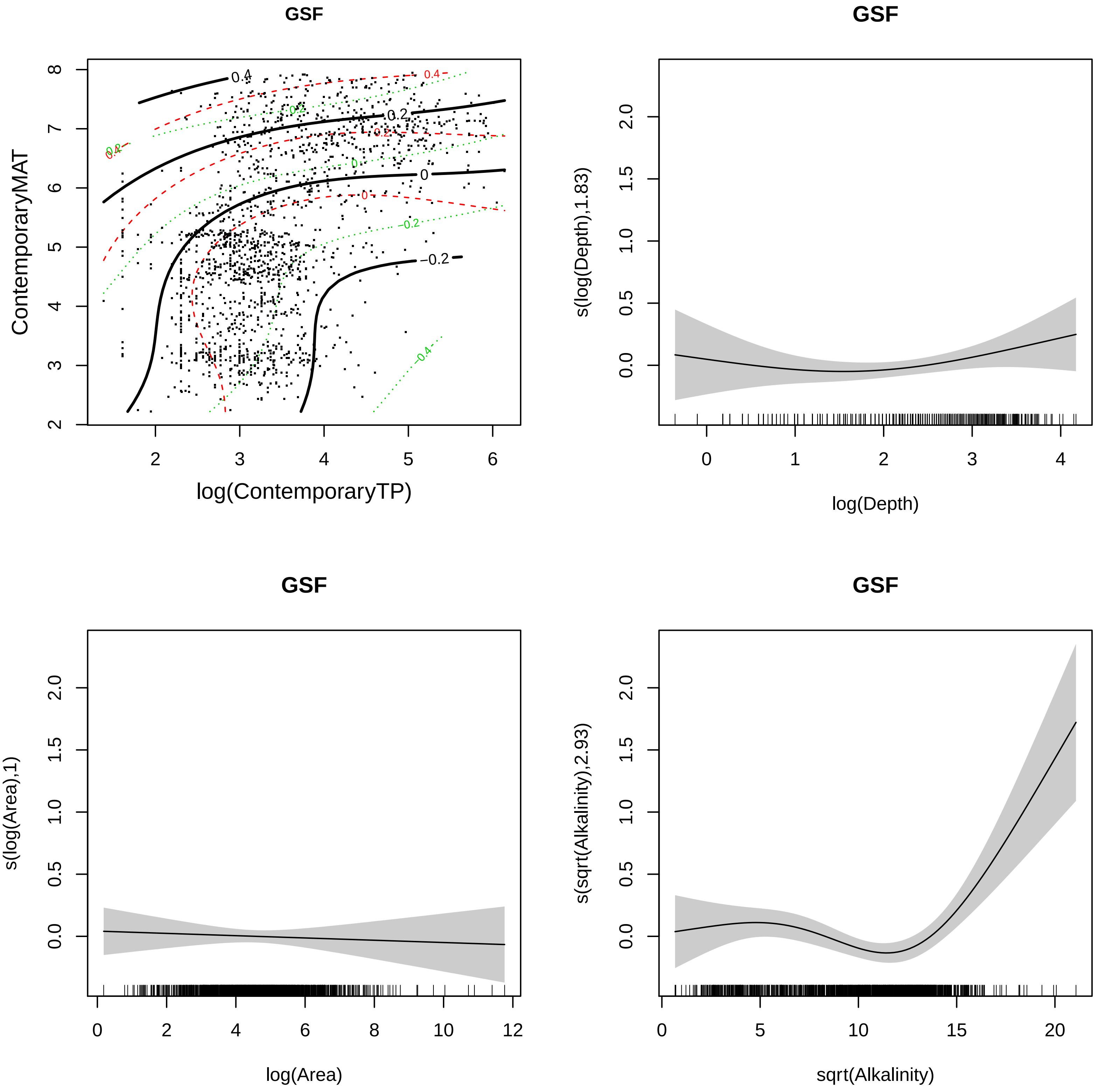
GSF
ContemporaryMAT
log(ContemporaryTP)
0.4
0.2
0
−0.2
−0.4
GSF
s(log(Depth),1.83)
log(Depth)
GSF
s(log(Area),1)
log(Area)
GSF
s(sqrt(Alkalinity),2.93)
sqrt(Alkalinity)

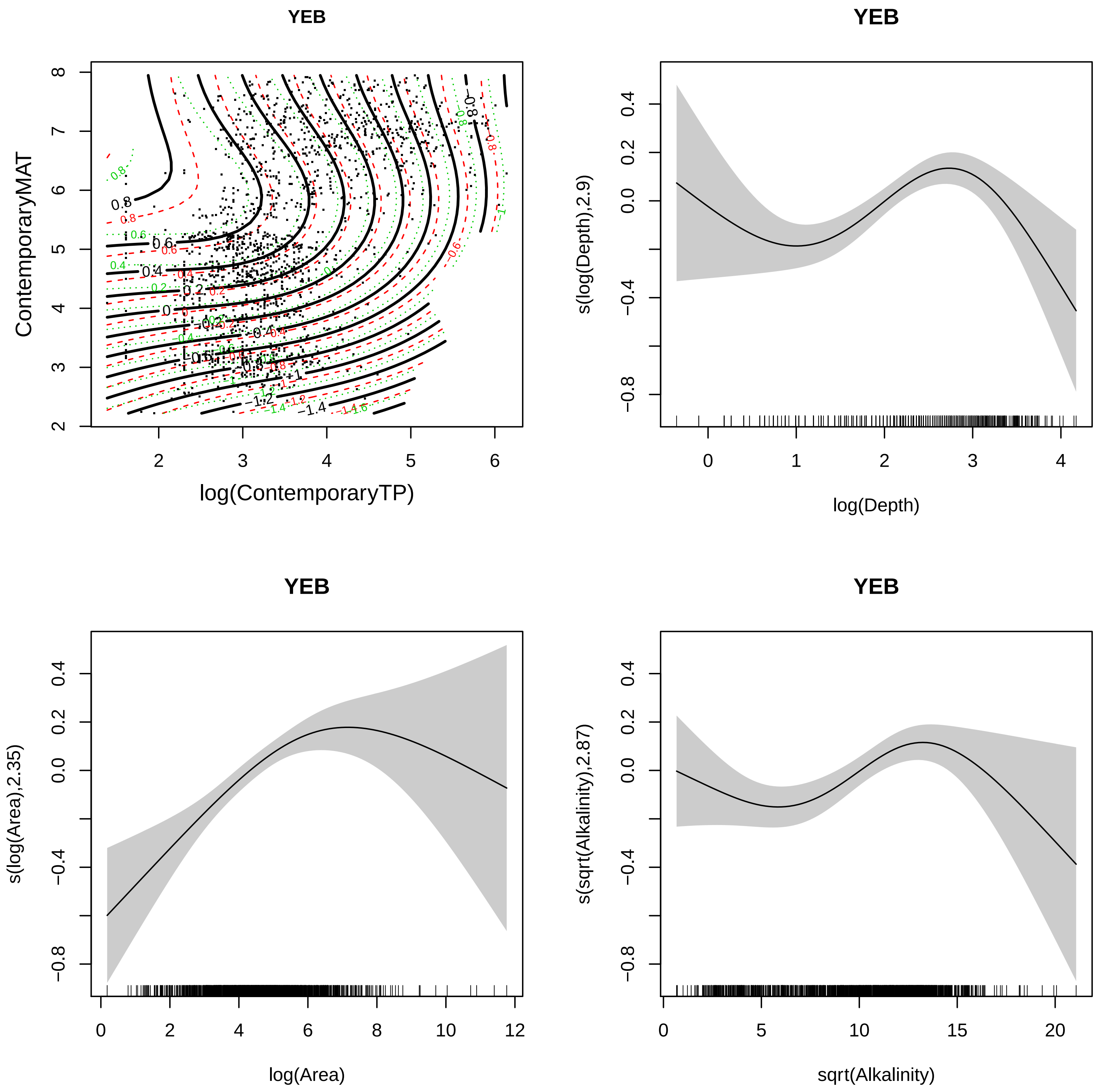
YEB
ContemporaryMAT
log(ContemporaryTP)
YEB
s(log(Depth),2.9)
log(Depth)
YEB
s(log(Area),2.35)
log(Area)
YEB
s(sqrt(Alkalinity),2.87)
sqrt(Alkalinity)

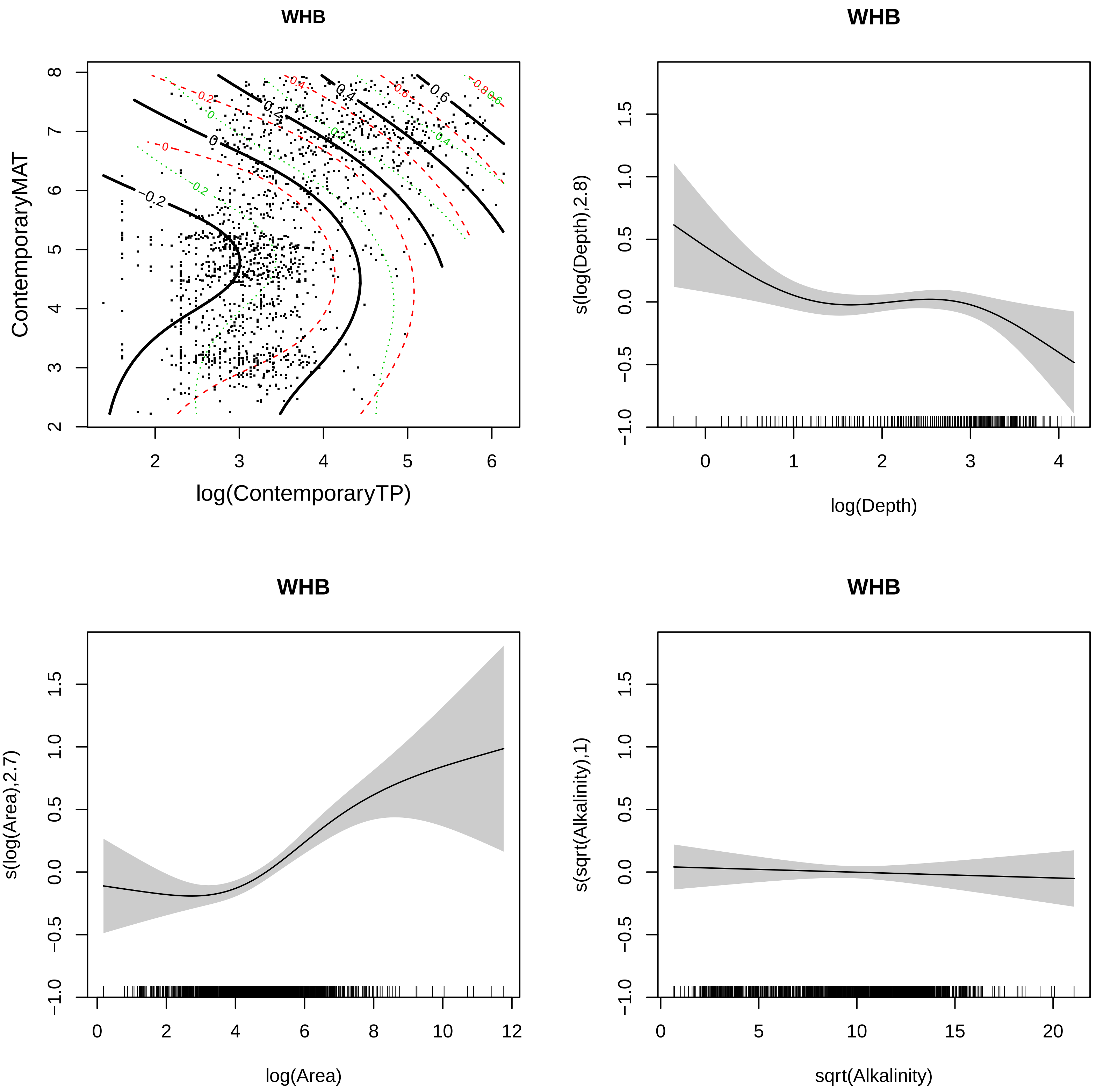
WHB
ContemporaryMAT
log(ContemporaryTP)
-0.2
0
0.2
0.4
0.6
WHB
s(log(Depth),2.8)
log(Depth)
WHB
s(log(Area),2.7)
log(Area)
WHB
s(sqrt(Alkalinity),1)
sqrt(Alkalinity)

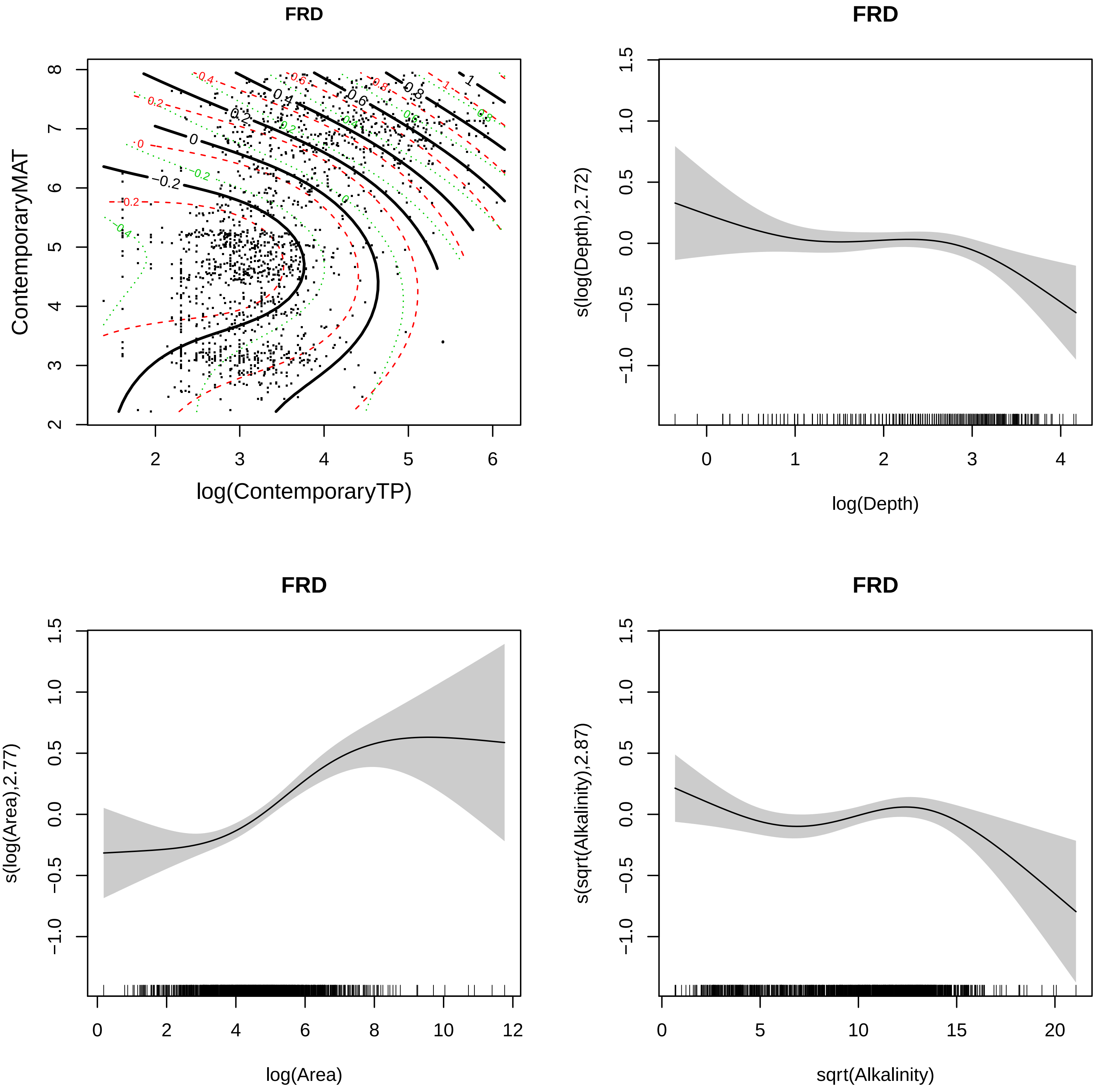

FRD
ContemporaryMAT
log(ContemporaryTP)
FRD
s(log(Depth),2.72)
log(Depth)
FRD
s(log(Area),2.77)
log(Area)
FRD
s(sqrt(Alkalinity),2.87)
sqrt(Alkalinity)

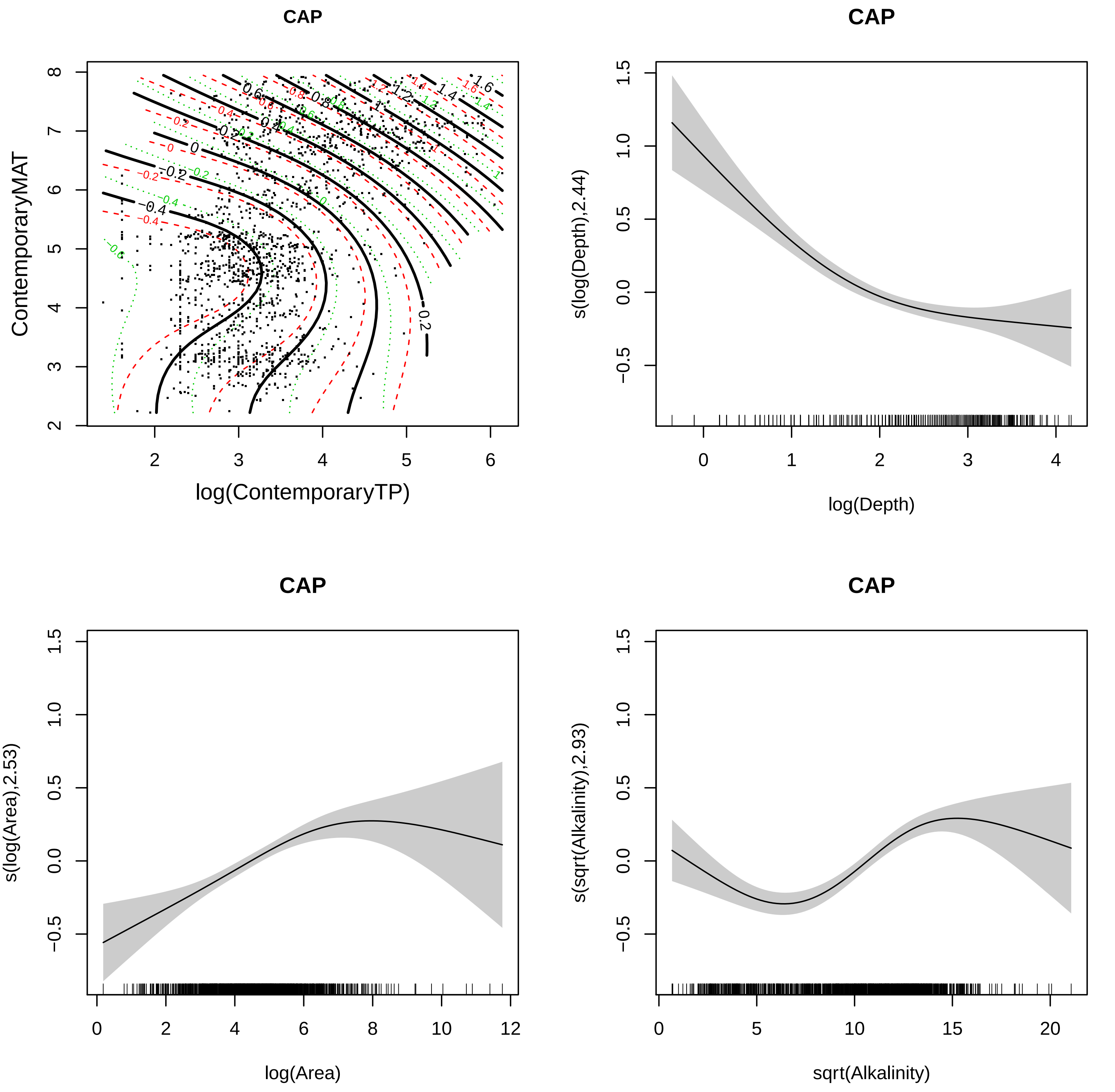
CAP
ContemporaryMAT
log(ContemporaryTP)
2
3
4
5
6
7
8
CAP
s(log(Depth),2.44)
log(Depth)
−0.5
0.0
0.5
1.0
1.5
0
1
2
3
4
CAP
s(log(Area),2.53)
log(Area)
−0.5
0.0
0.5
1.0
1.5
0
2
4
6
8
10
12
CAP
s(sqrt(Alkalinity),2.93)
sqrt(Alkalinity)
−0.5
0.0
0.5
1.0
1.5
0
5
10
15
20

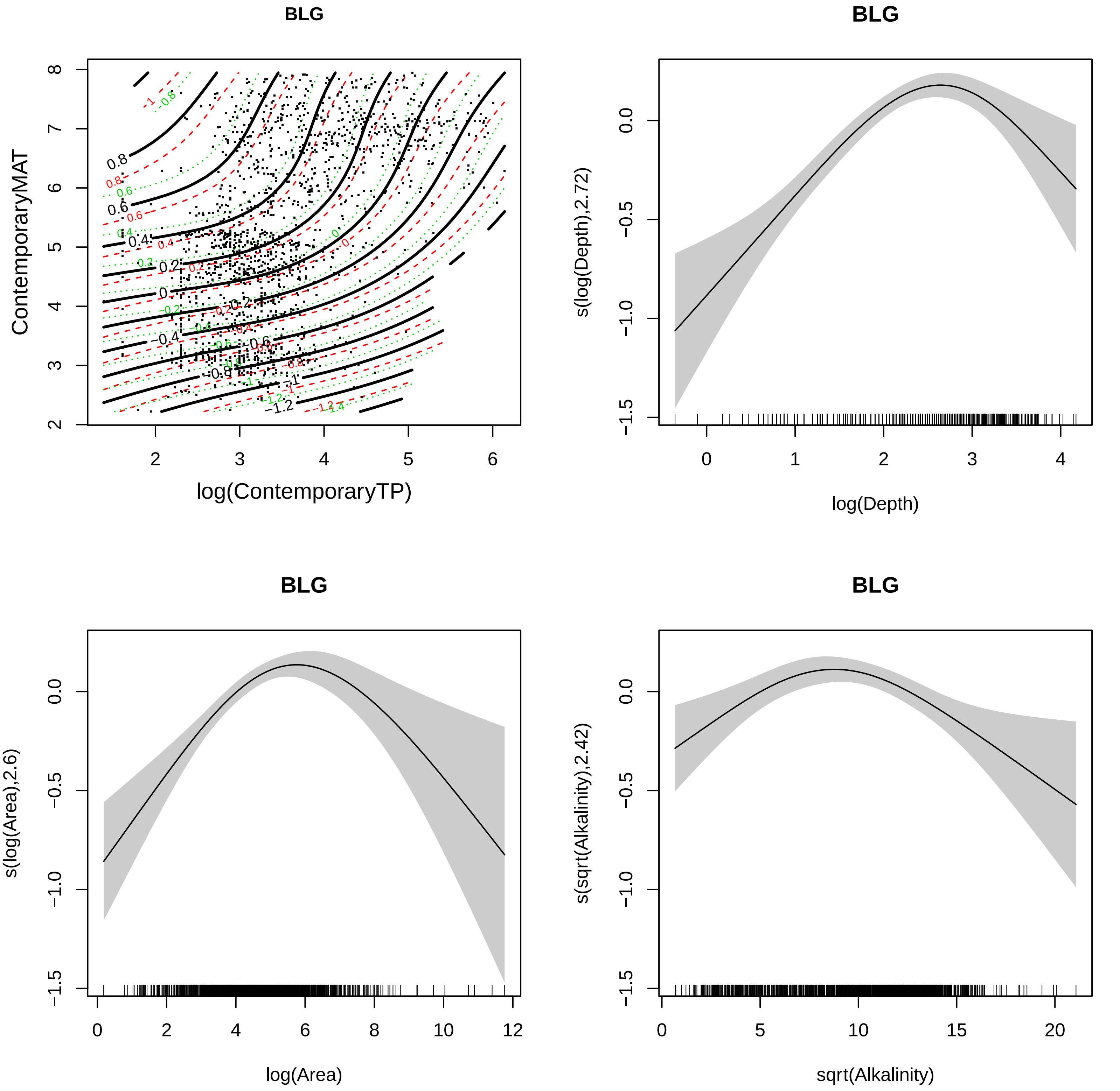
BLG
ContemporaryMAT
log(ContemporaryTP)
BLG
s(log(Depth),2.72)
log(Depth)
BLG
s(log(Area),2.6)
log(Area)
BLG
s(sqrt(Alkalinity),2.42)
sqrt(Alkalinity)

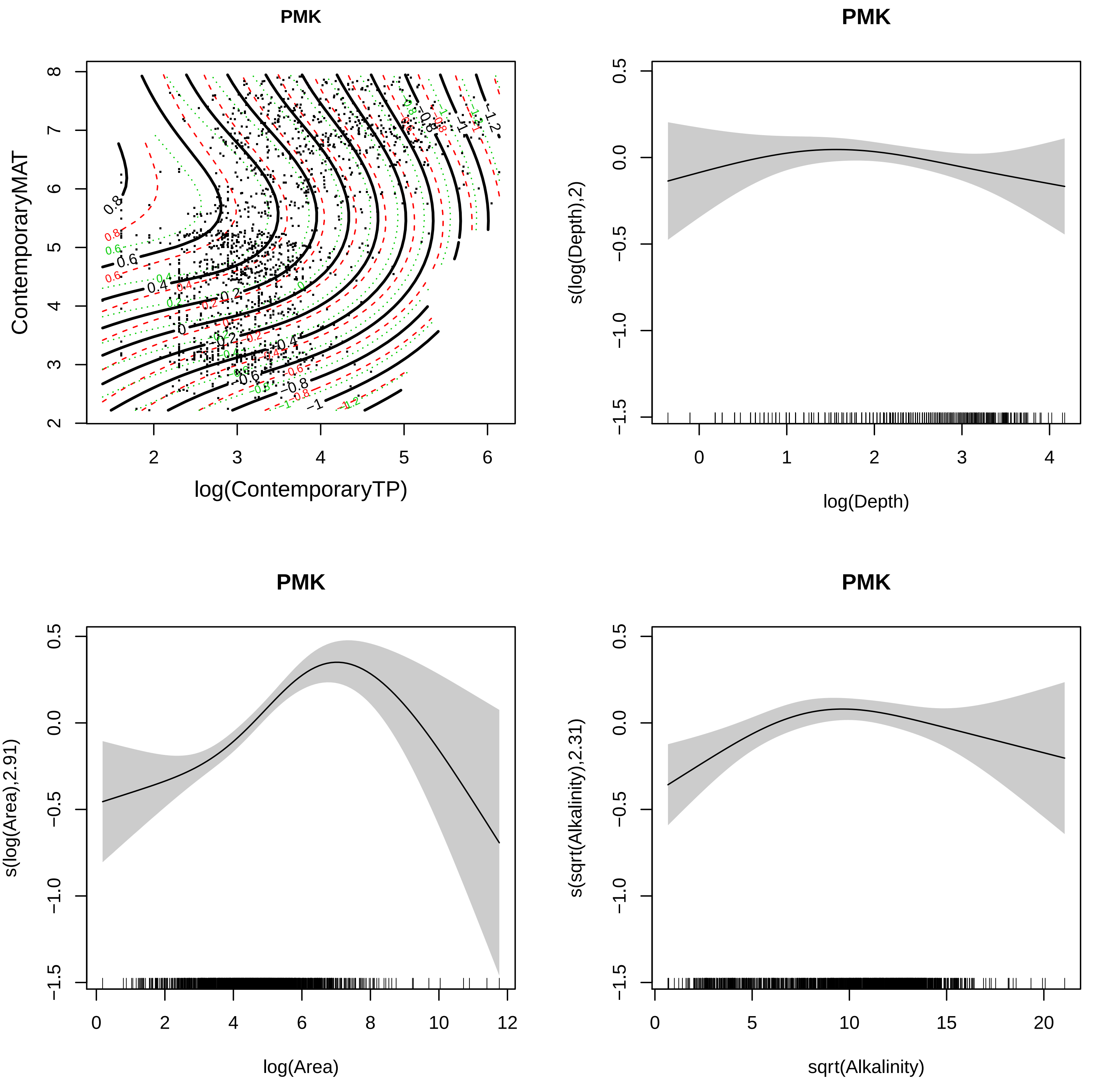
PMK
ContemporaryMAT
log(ContemporaryTP)
2 3 4 5 6 7 8
2 3 4 5 6
PMK
s(log(Depth),2)
log(Depth)
−1.5 −1.0 −0.5 0.0 0.5
0 1 2 3 4
PMK
s(log(Area),2.91)
log(Area)
−1.5 −1.0 −0.5 0.0 0.5
0 2 4 6 8 10 12
PMK
s(sqrt(Alkalinity),2.31)
sqrt(Alkalinity)
−1.5 −1.0 −0.5 0.0 0.5
0 5 10 15 20

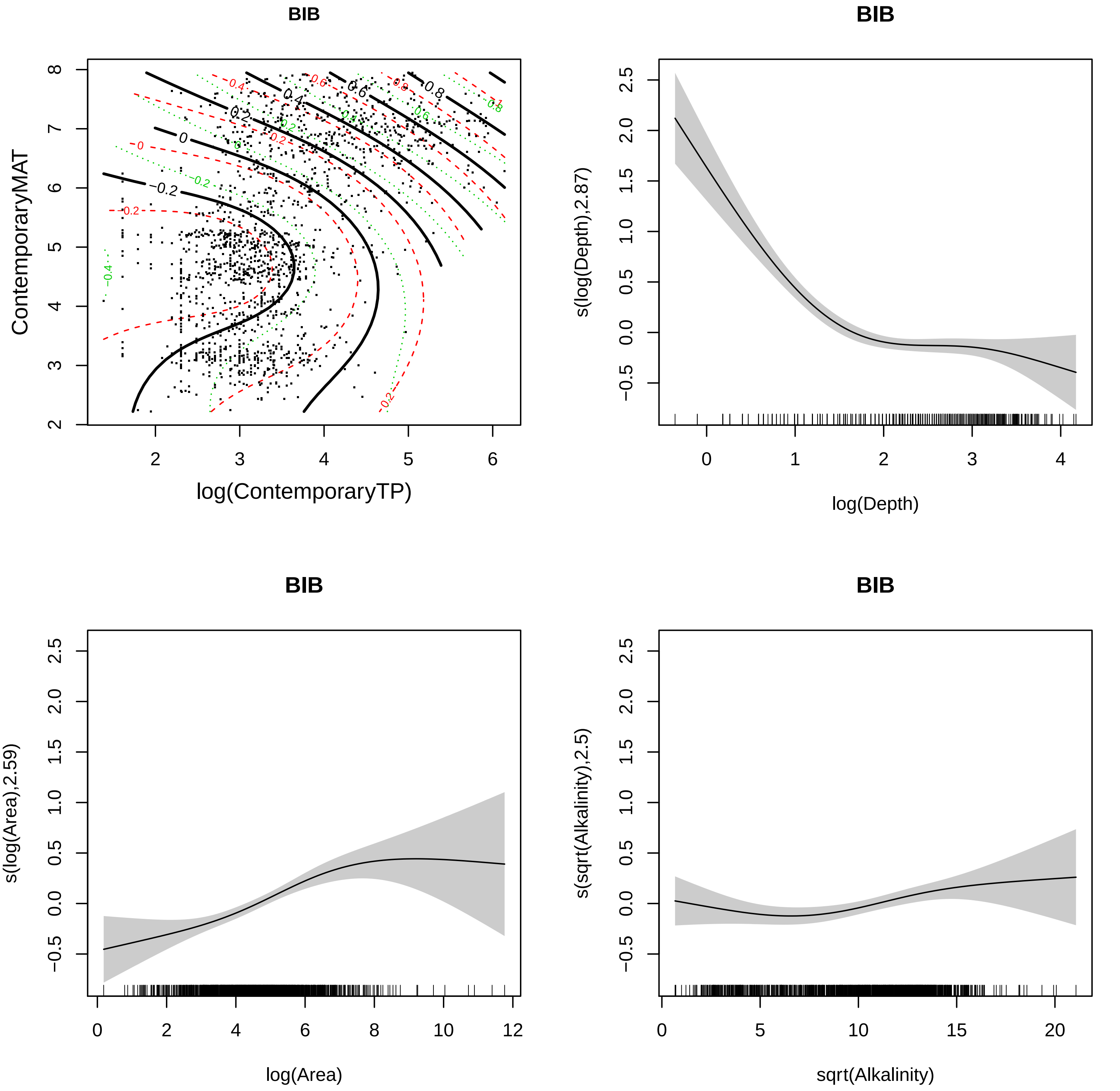
BIB
ContemporaryMAT
log(ContemporaryTP)
BIB
s(log(Depth),2.87)
log(Depth)
BIB
s(log(Area),2.59)
log(Area)
BIB
s(sqrt(Alkalinity),2.5)
sqrt(Alkalinity)

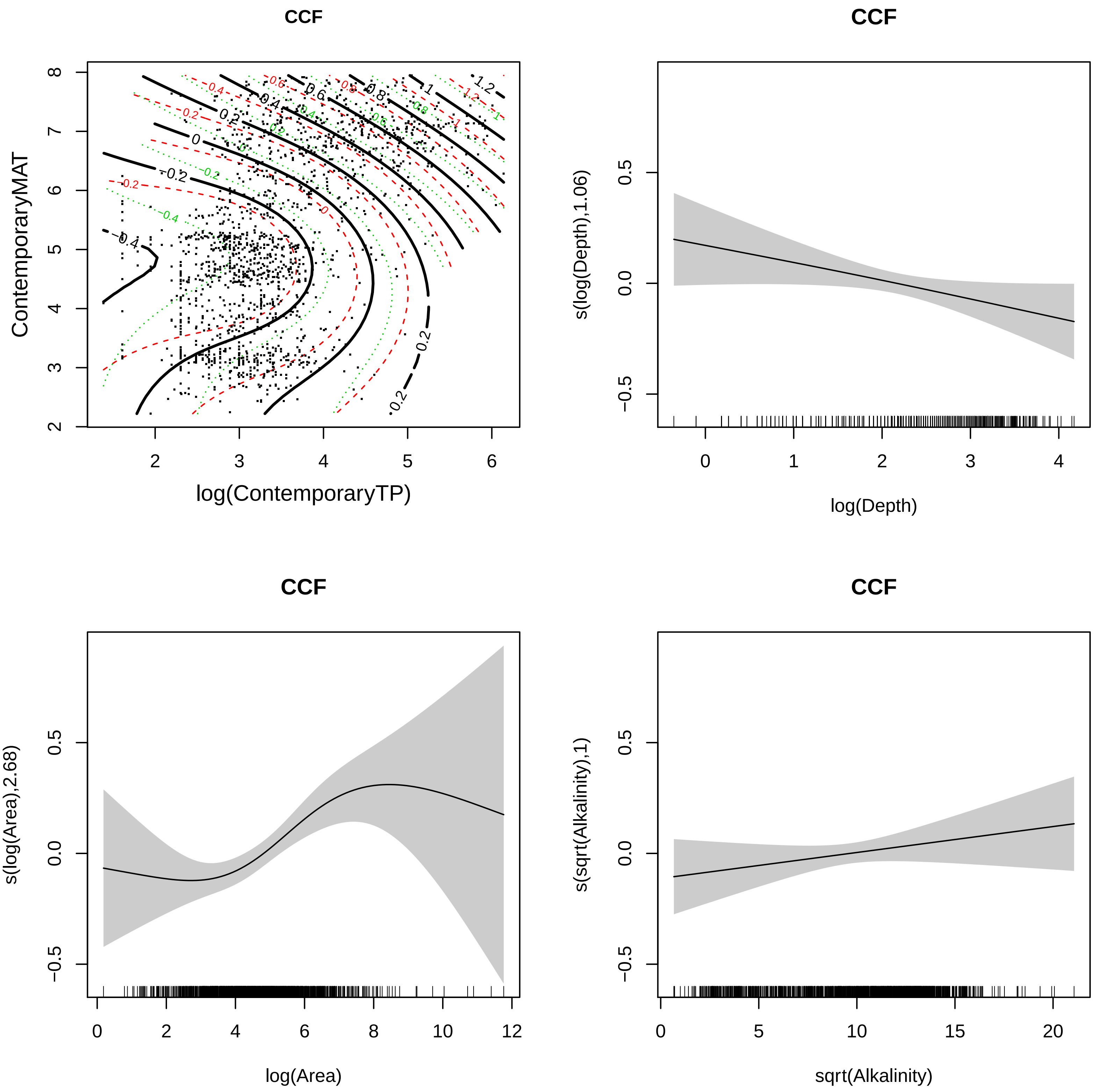
CCF
ContemporaryMAT
2
3
4
5
6
7
8
log(ContemporaryTP)
2
3
4
5
6
CCF
s(log(Depth),1.06)
−0.5
0.0
0.5
log(Depth)
0
1
2
3
4
CCF
s(log(Area),2.68)
−0.5
0.0
0.5
log(Area)
0
2
4
6
8
10
12
CCF
s(sqrt(Alkalinity),1)
−0.5
0.0
0.5
sqrt(Alkalinity)
0
5
10
15
20

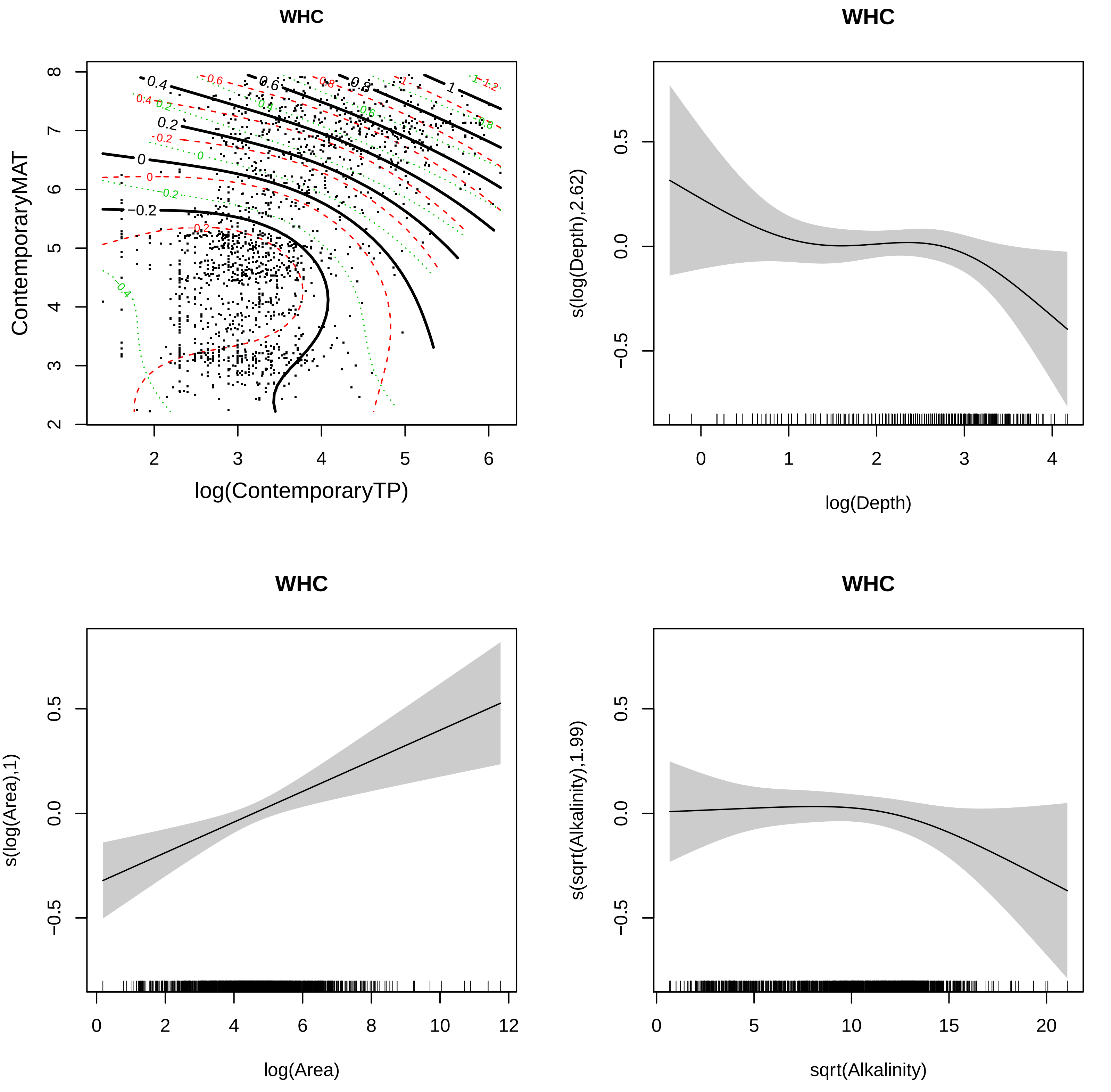

WHC
ContemporaryMAT
log(ContemporaryTP)
-0.2
0
0.2
0.4
0.6
0.8
1
WHC
s(log(Depth),2.62)
log(Depth)
WHC
s(log(Area),1)
log(Area)
WHC
s(sqrt(Alkalinity),1.99)
sqrt(Alkalinity)

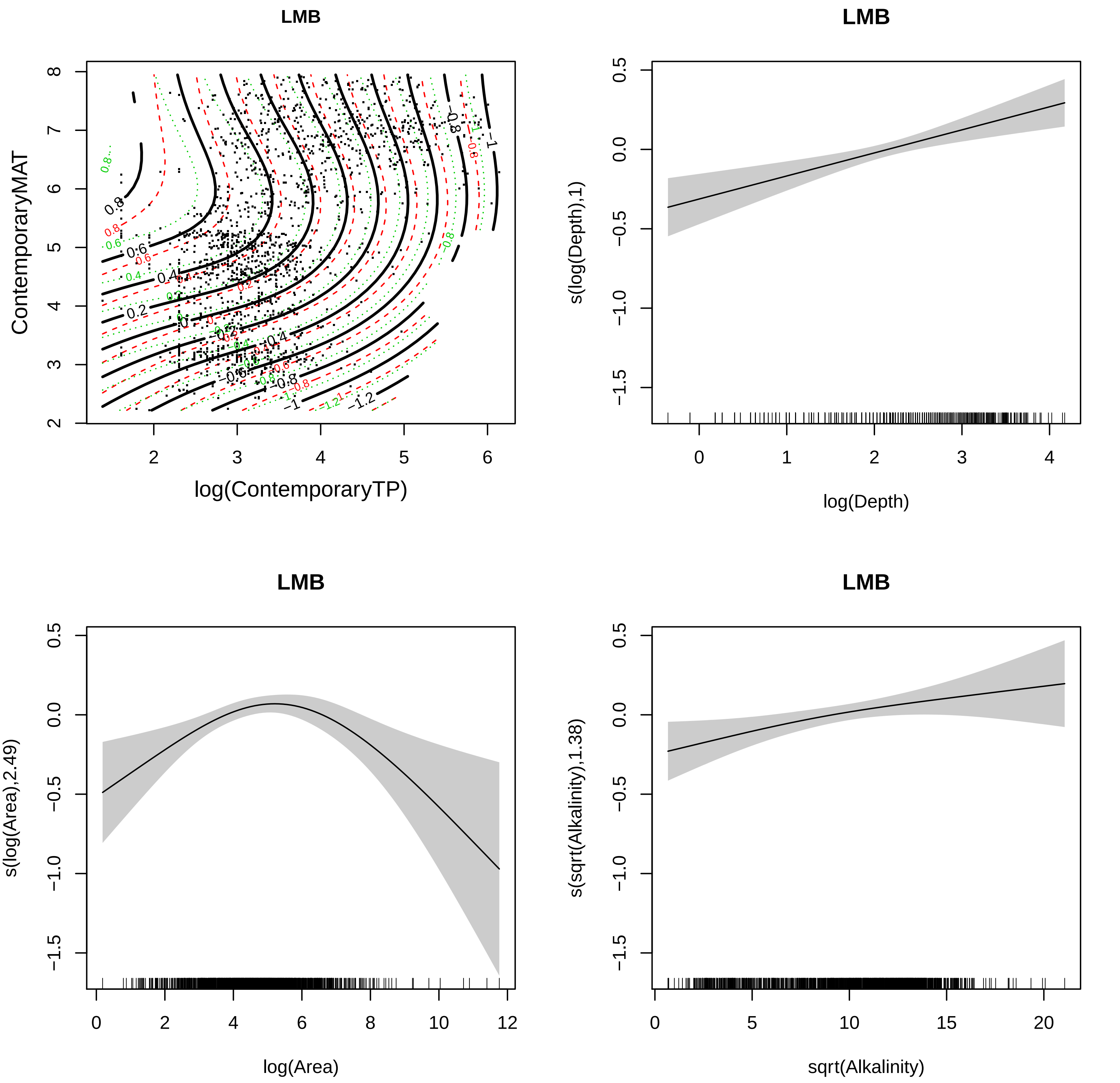

LMB
ContemporaryMAT
log(ContemporaryTP)
LMB
s(log(Depth),1)
log(Depth)
LMB
s(log(Area),2.49)
log(Area)
LMB
s(sqrt(Alkalinity),1.38)
sqrt(Alkalinity)

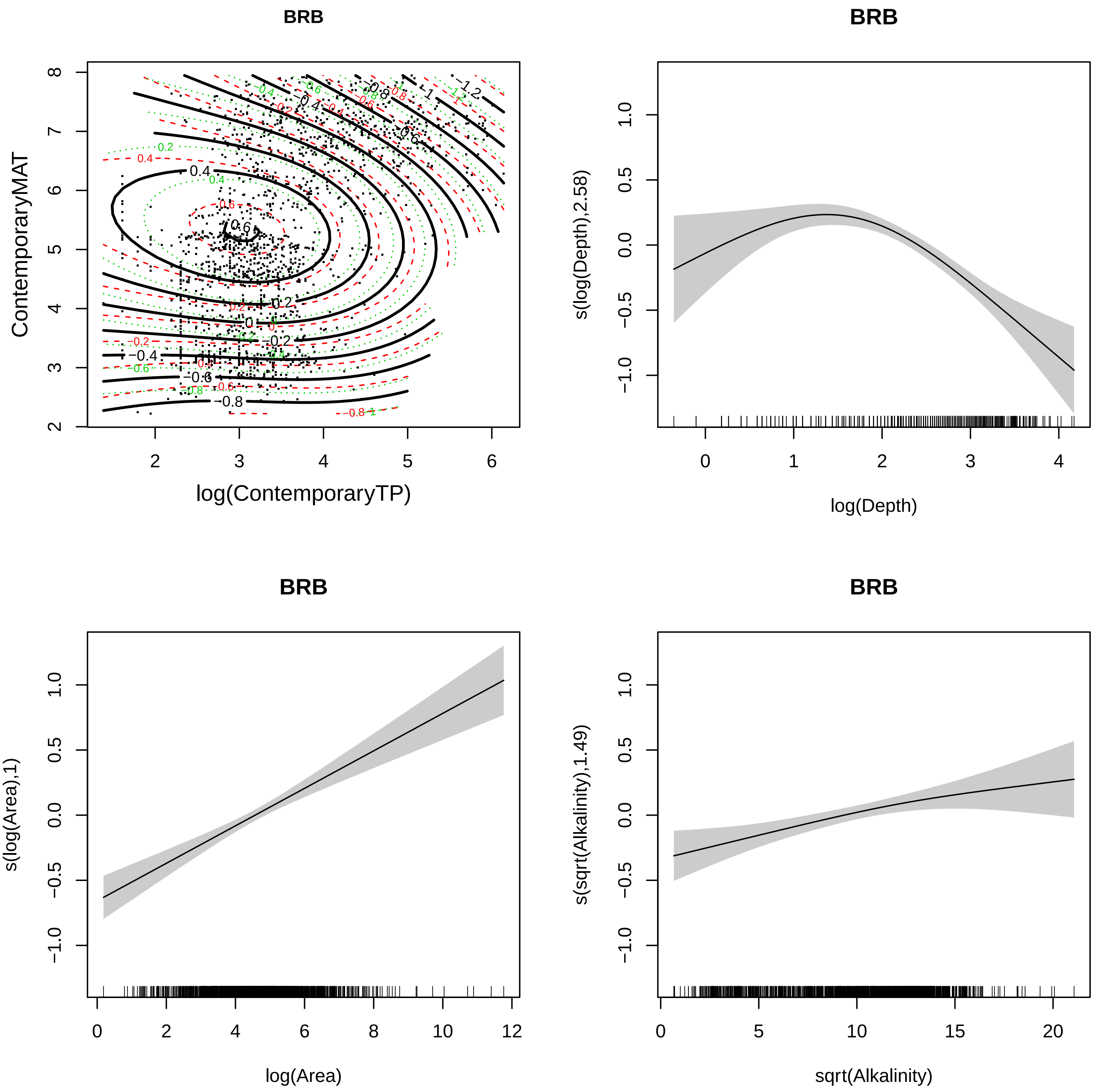
BRB
ContemporaryMAT
log(ContemporaryTP)
BRB
s(log(Depth),2.58)
log(Depth)
BRB
s(log(Area),1)
log(Area)
BRB
s(sqrt(Alkalinity),1.49)
sqrt(Alkalinity)

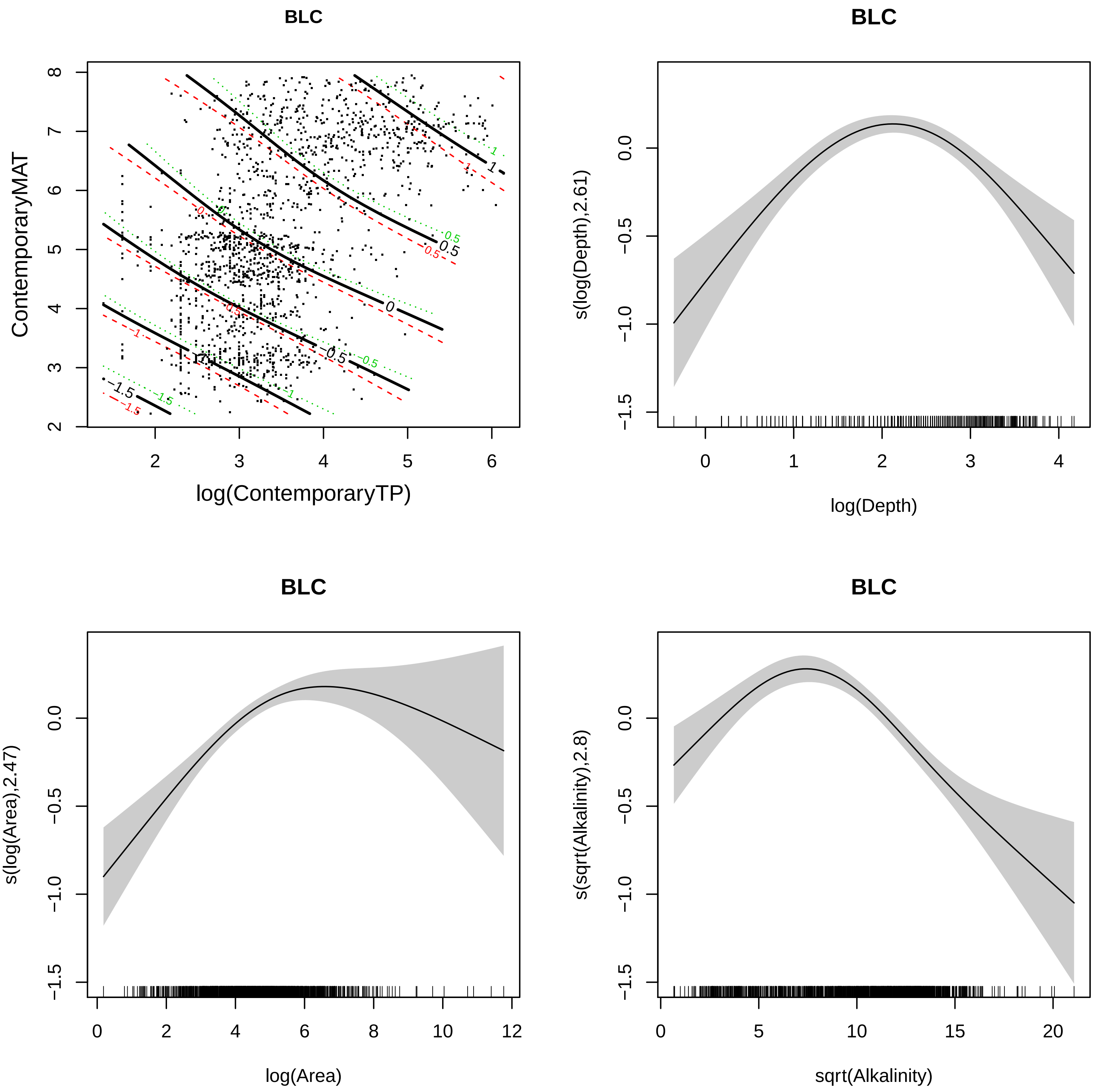
BLC
ContemporaryMAT
log(ContemporaryTP)
BLC
s(log(Depth),2.61)
log(Depth)
BLC
s(log(Area),2.47)
log(Area)
BLC
s(sqrt(Alkalinity),2.8)
sqrt(Alkalinity)

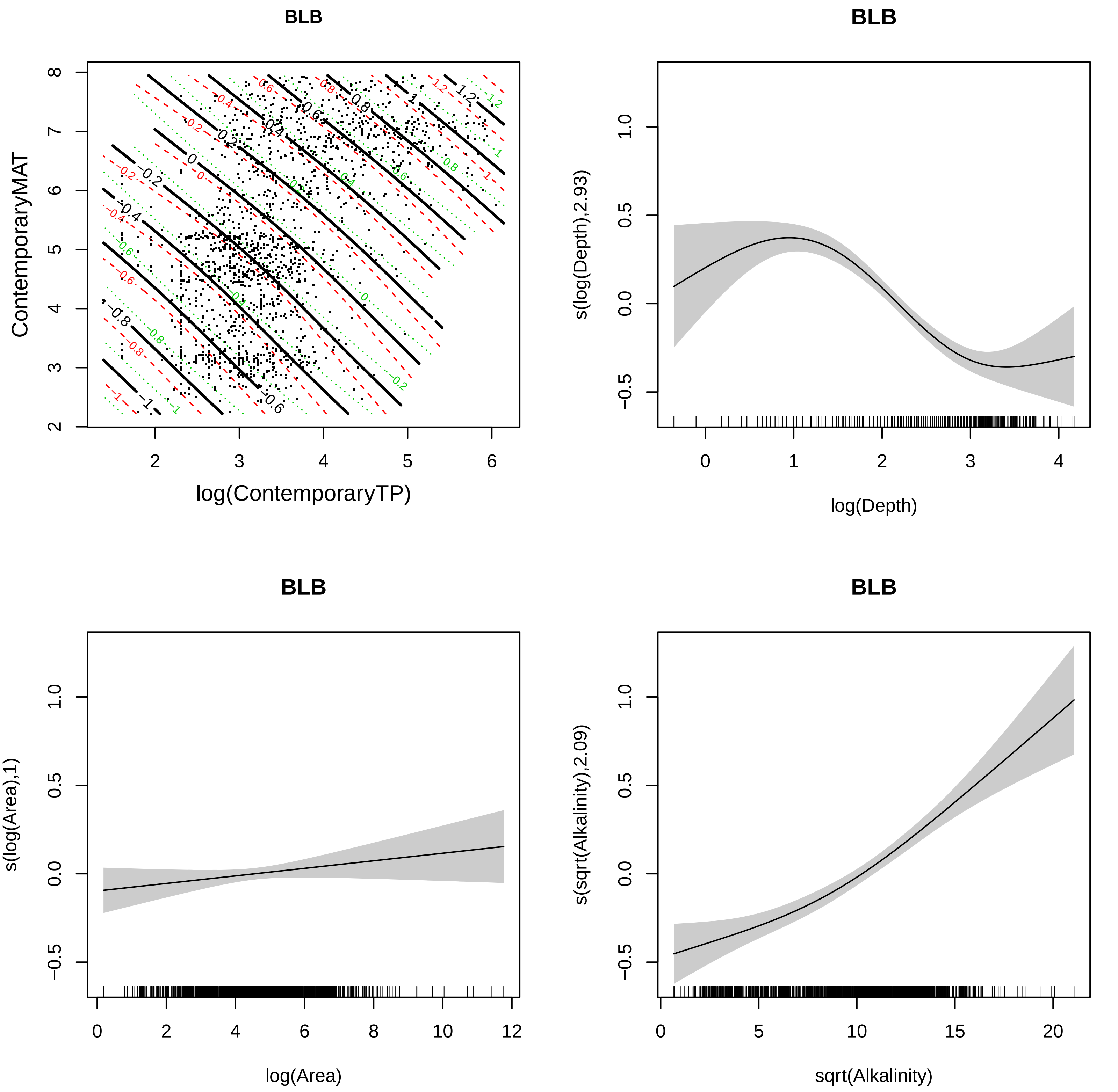
BLB
ContemporaryMAT
log(ContemporaryTP)
BLB
s(log(Depth),2.93)
log(Depth)
BLB
s(log(Area),1)
log(Area)
BLB
s(sqrt(Alkalinity),2.09)
sqrt(Alkalinity)

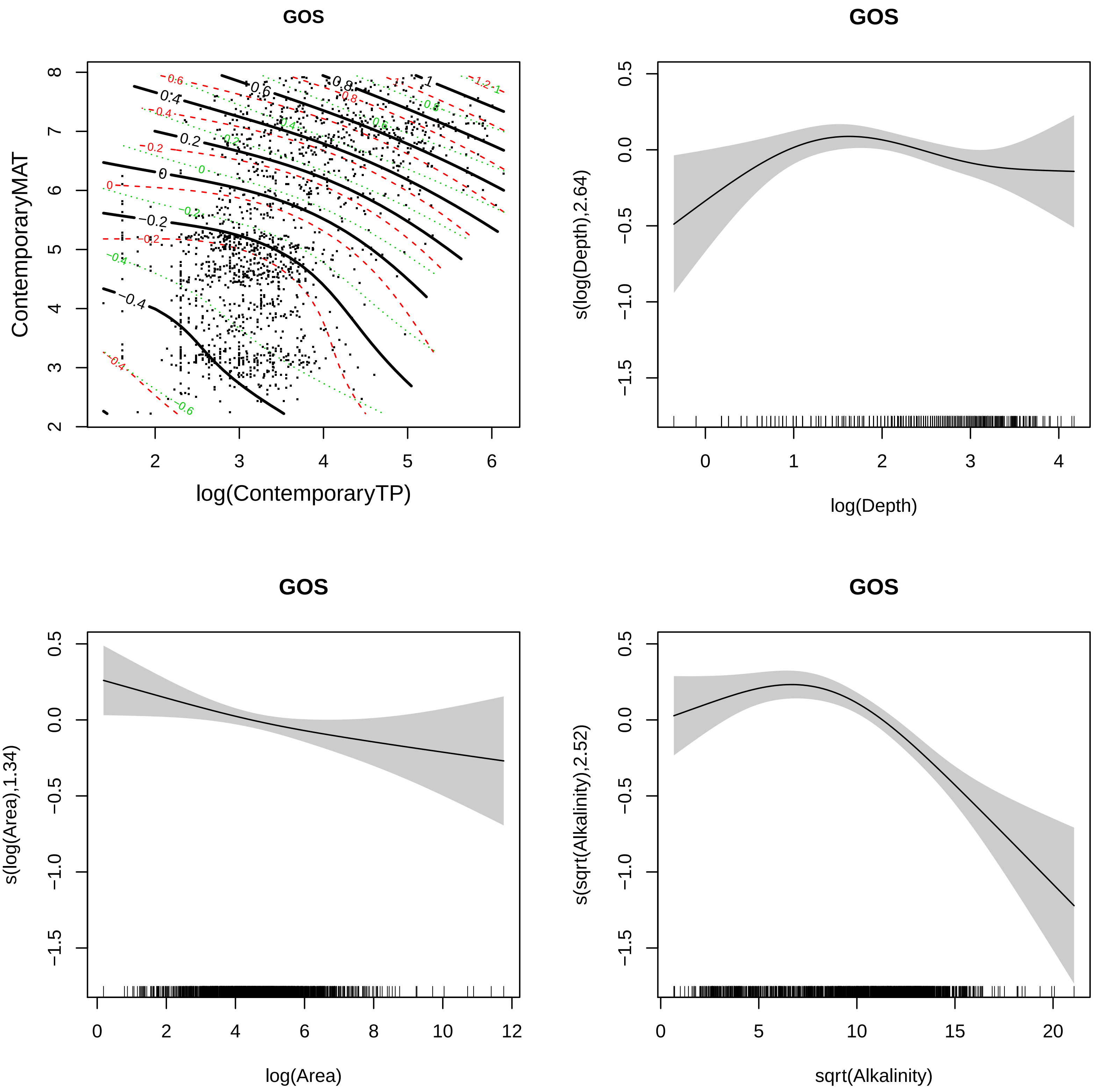
GOS
ContemporaryMAT
log(ContemporaryTP)
GOS
s(log(Depth),2.64)
log(Depth)
GOS
s(log(Area),1.34)
log(Area)
GOS
s(sqrt(Alkalinity),2.52)
sqrt(Alkalinity)

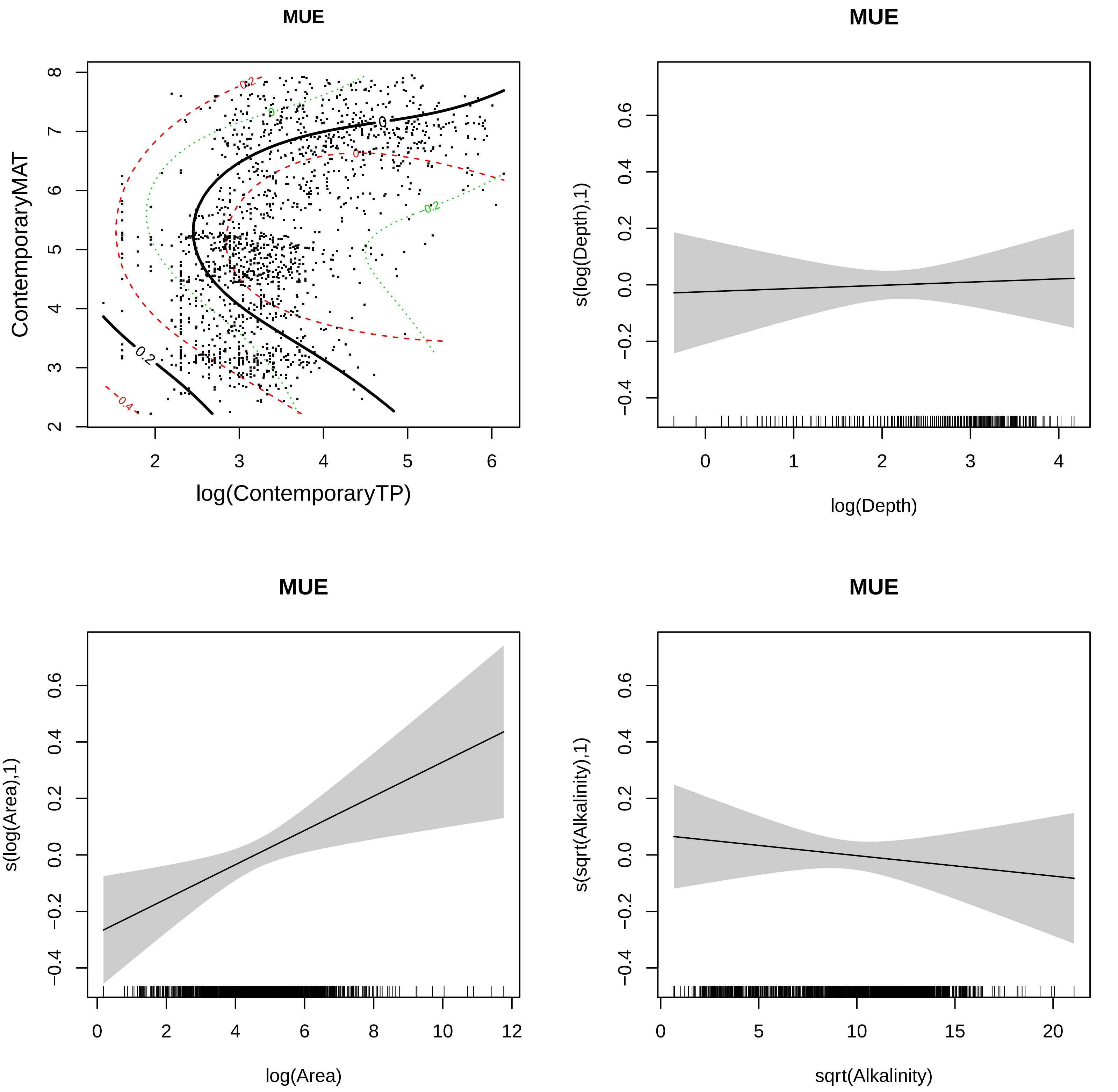
MUE
ContemporaryMAT
log(ContemporaryTP)
MUE
s(log(Depth),1)
log(Depth)
MUE
s(log(Area),1)
log(Area)
MUE
s(sqrt(Alkalinity),1)
sqrt(Alkalinity)

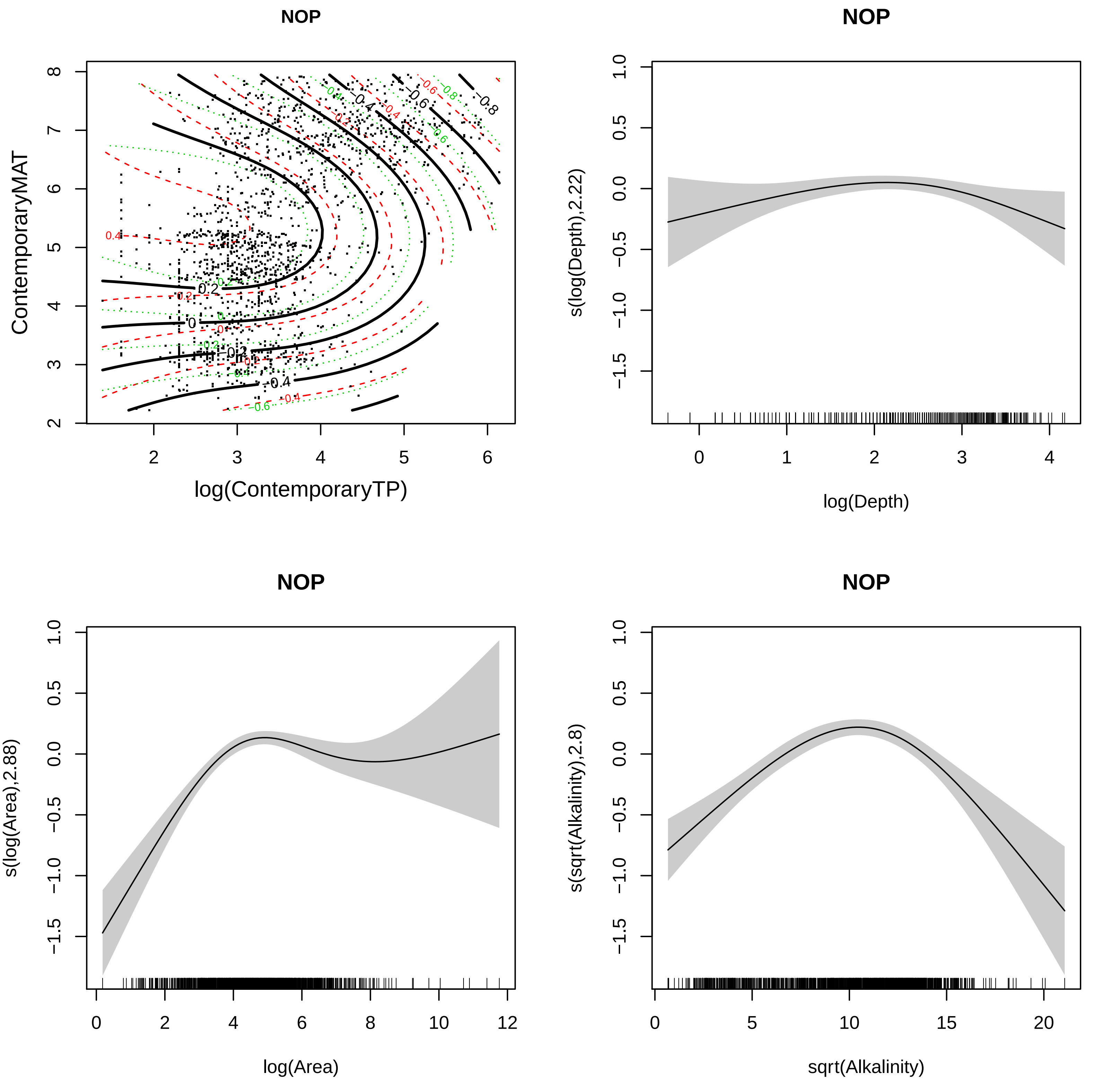
NOP
ContemporaryMAT
log(ContemporaryTP)
NOP
s(log(Depth),2.22)
log(Depth)
NOP
s(log(Area),2.88)
log(Area)
NOP
s(sqrt(Alkalinity),2.8)
sqrt(Alkalinity)

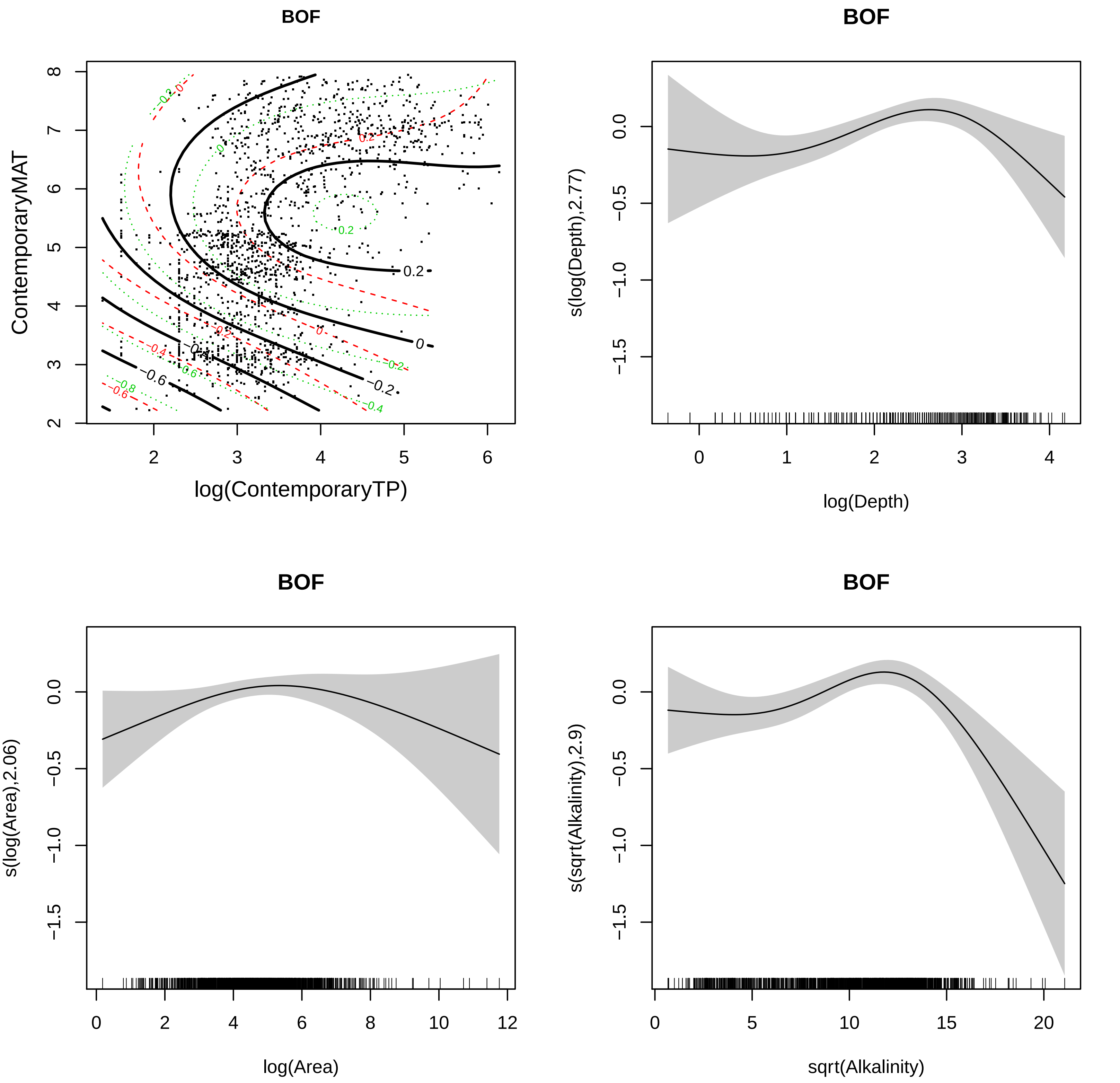
BOF
ContemporaryMAT
log(ContemporaryTP)
2
3
4
5
6
7
8
-0.6
-0.4
-0.2
0
0.2
0.4
0.6
0.8
BOF
s(log(Depth),2.77)
log(Depth)
0.0
−0.5
−1.0
−1.5
0
1
2
3
4
BOF
s(log(Area),2.06)
log(Area)
0.0
−0.5
−1.0
−1.5
0
2
4
6
8
10
12
BOF
s(sqrt(Alkalinity),2.9)
sqrt(Alkalinity)
0.0
−0.5
−1.0
−1.5
0
5
10
15
20

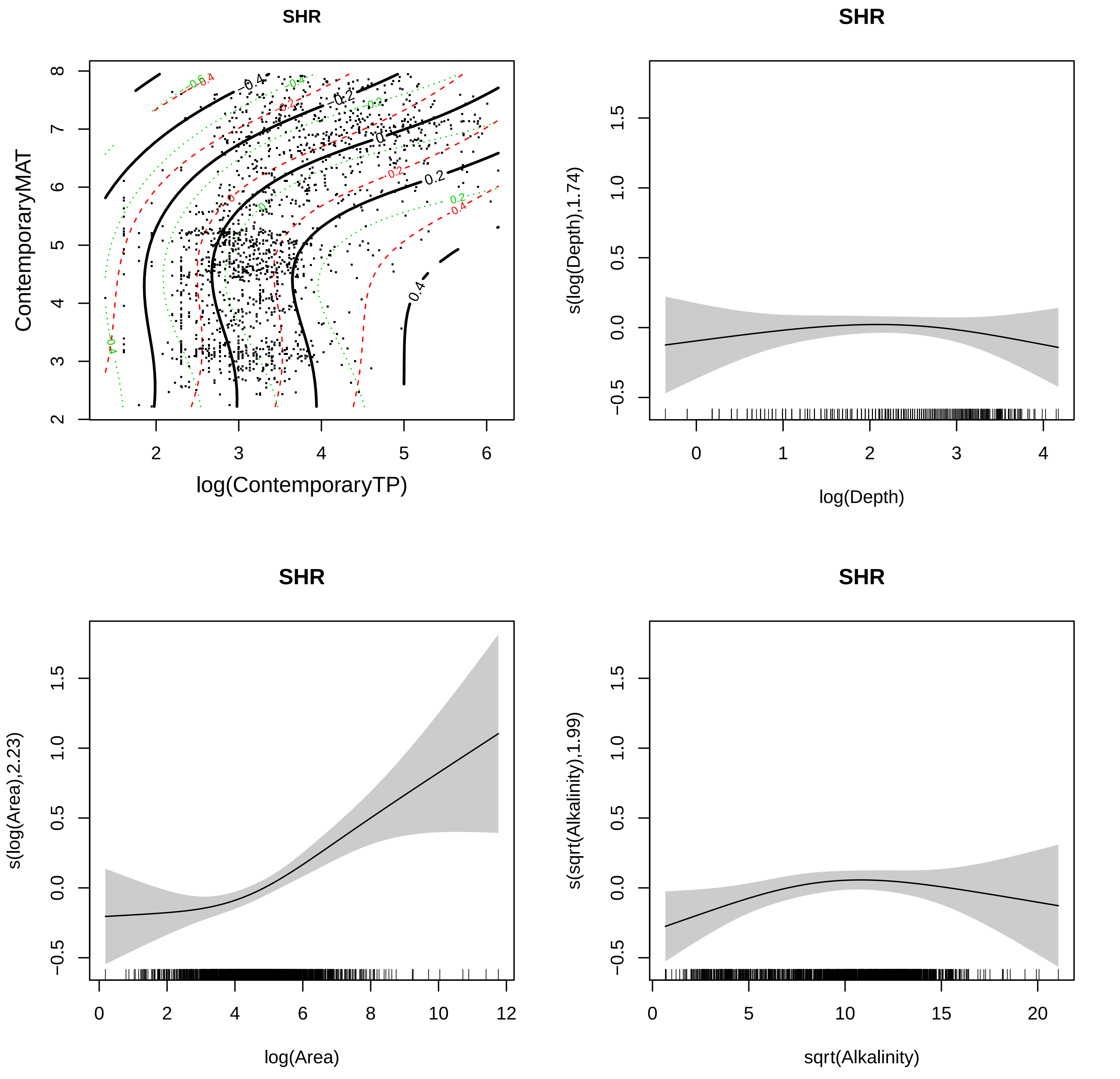

SHR
ContemporaryMAT
log(ContemporaryTP)
SHR
s(log(Depth),1.74)
log(Depth)
SHR
s(log(Area),2.23)
log(Area)
SHR
s(sqrt(Alkalinity),1.99)
sqrt(Alkalinity)

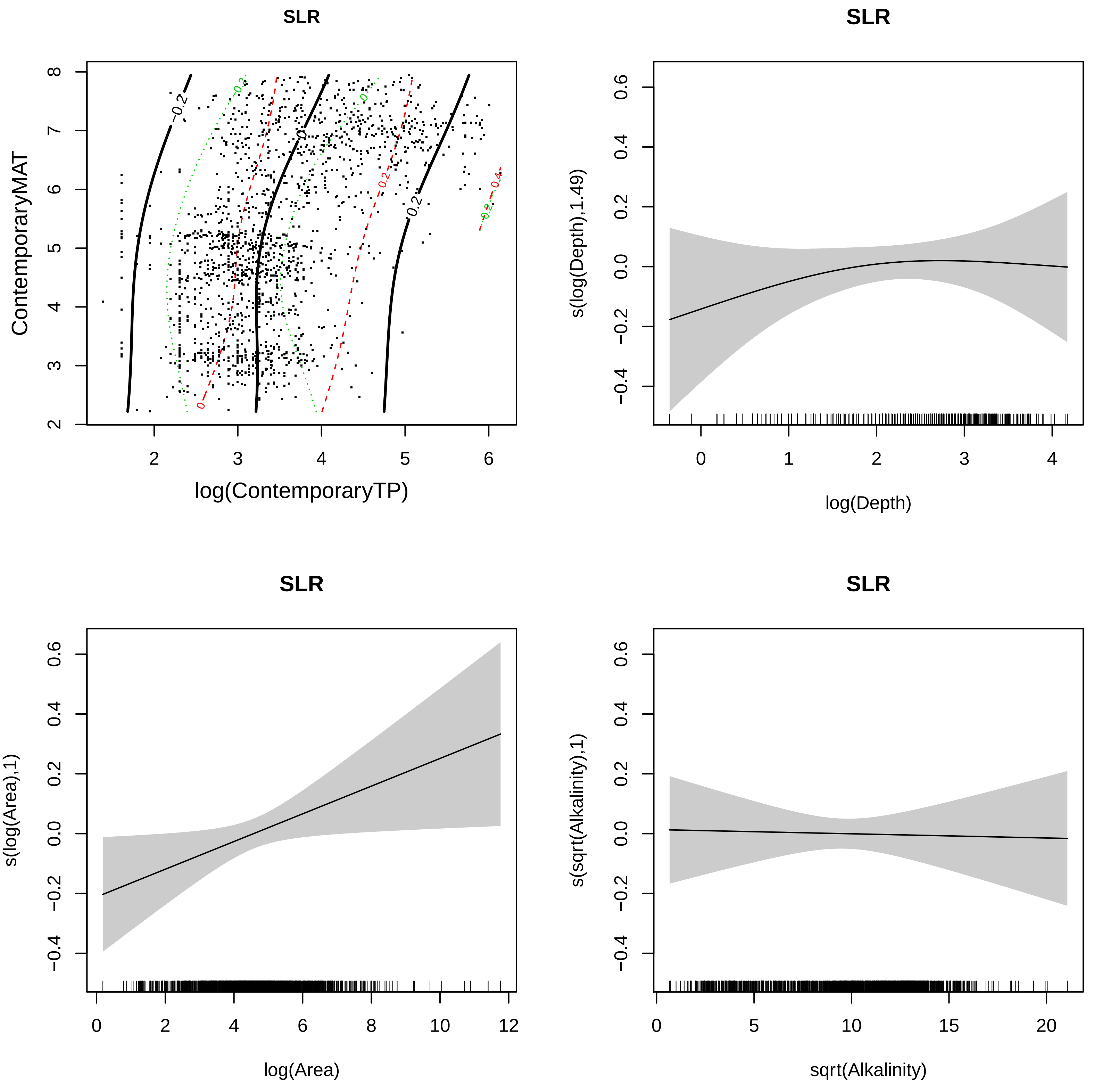

SLR
ContemporaryMAT
log(ContemporaryTP)
−0.2
0
0.2
0.4
2
3
4
5
6
7
8
SLR
s(log(Depth),1.49)
log(Depth)
−0.4
−0.2
0.0
0.2
0.4
0.6
0
1
2
3
4
SLR
s(log(Area),1)
log(Area)
−0.4
−0.2
0.0
0.2
0.4
0.6
0
2
4
6
8
10
12
SLR
s(sqrt(Alkalinity),1)
sqrt(Alkalinity)
−0.4
−0.2
0.0
0.2
0.4
0.6
0
5
10
15
20

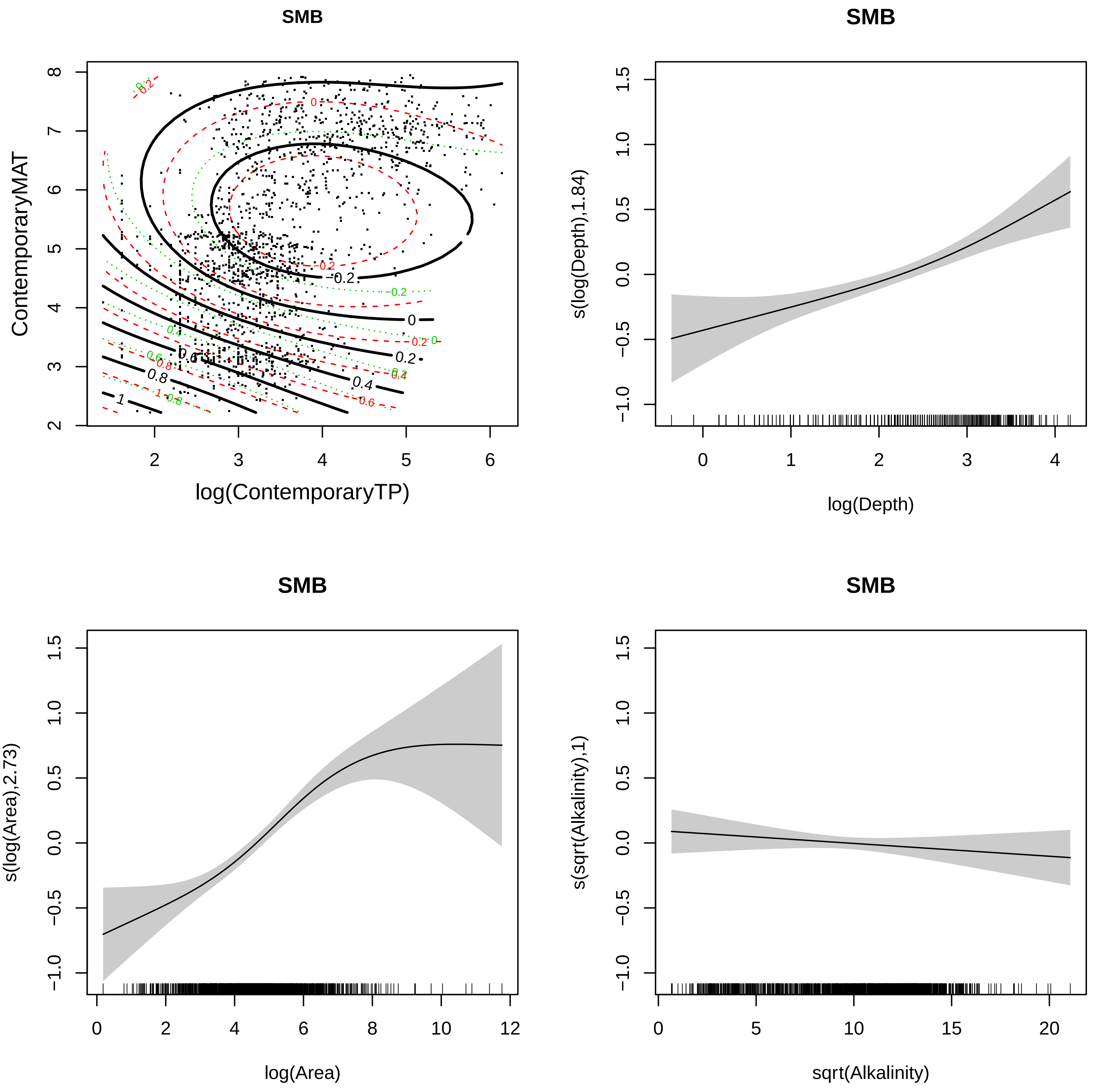
SMB
ContemporaryMAT
log(ContemporaryTP)
SMB
s(log(Depth),1.84)
log(Depth)
SMB
s(log(Area),2.73)
log(Area)
SMB
s(sqrt(Alkalinity),1)
sqrt(Alkalinity)

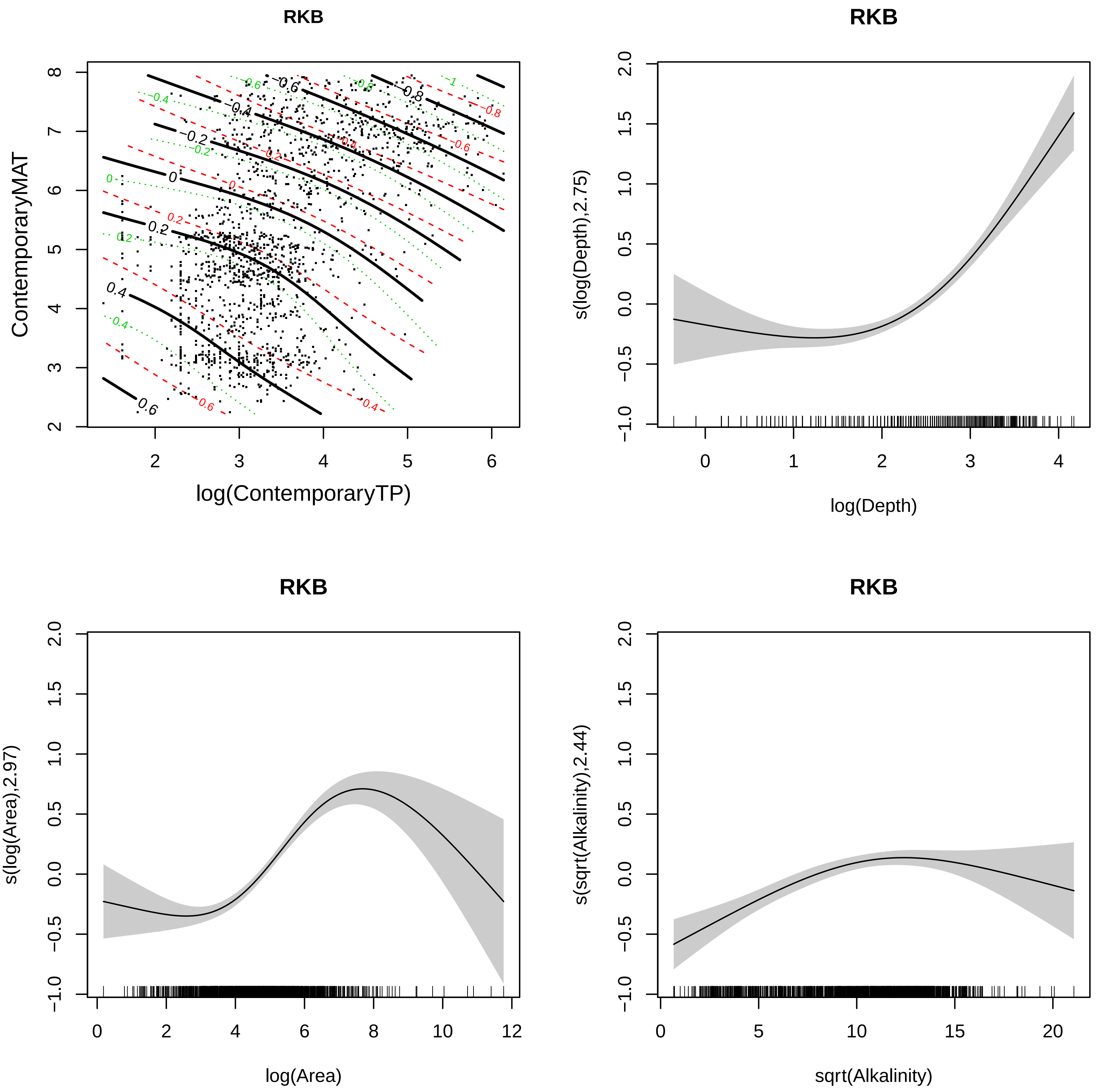
RKB
ContemporaryMAT
log(ContemporaryTP)
RKB
s(log(Depth),2.75)
log(Depth)
RKB
s(log(Area),2.97)
log(Area)
RKB
s(sqrt(Alkalinity),2.44)
sqrt(Alkalinity)

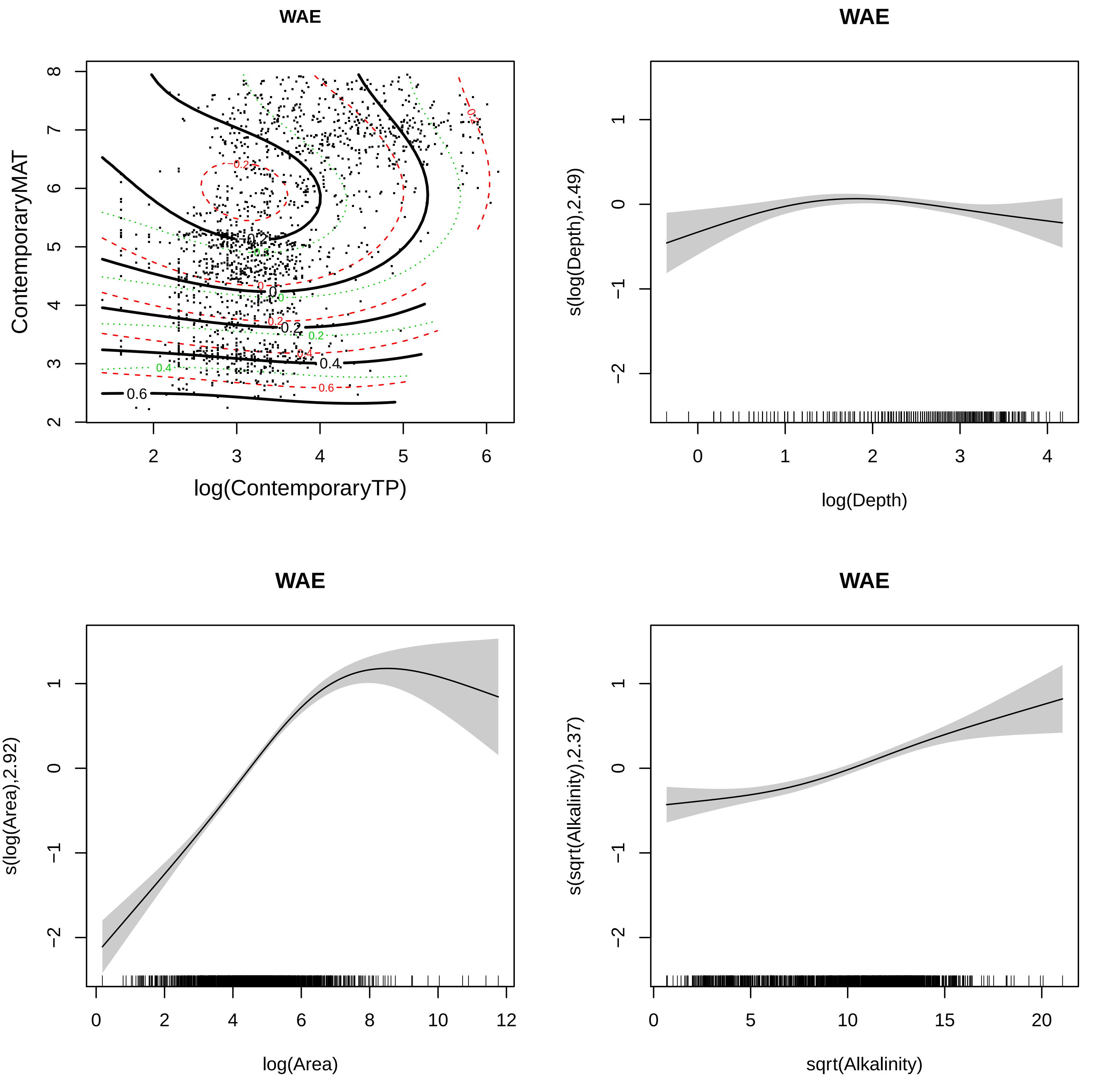
WAE
ContemporaryMAT
log(ContemporaryTP)
WAE
s(log(Depth),2.49)
log(Depth)
WAE
s(log(Area),2.92)
log(Area)
WAE
s(sqrt(Alkalinity),2.37)
sqrt(Alkalinity)

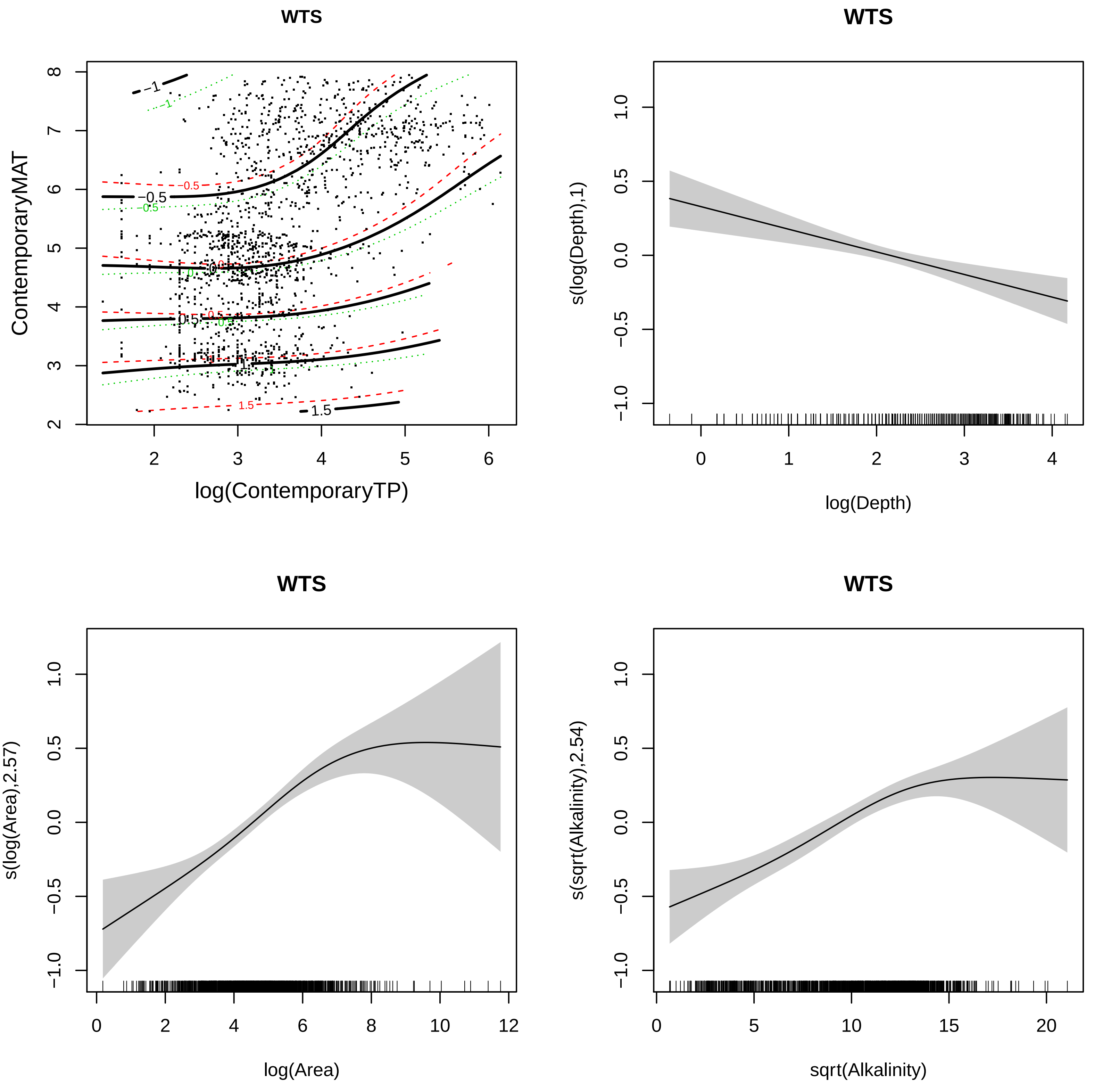

WTS
ContemporaryMAT
log(ContemporaryTP)
-1
-0.5
0.5
1.5
WTS
s(log(Depth),1)
log(Depth)
WTS
s(log(Area),2.57)
log(Area)
WTS
s(sqrt(Alkalinity),2.54)
sqrt(Alkalinity)

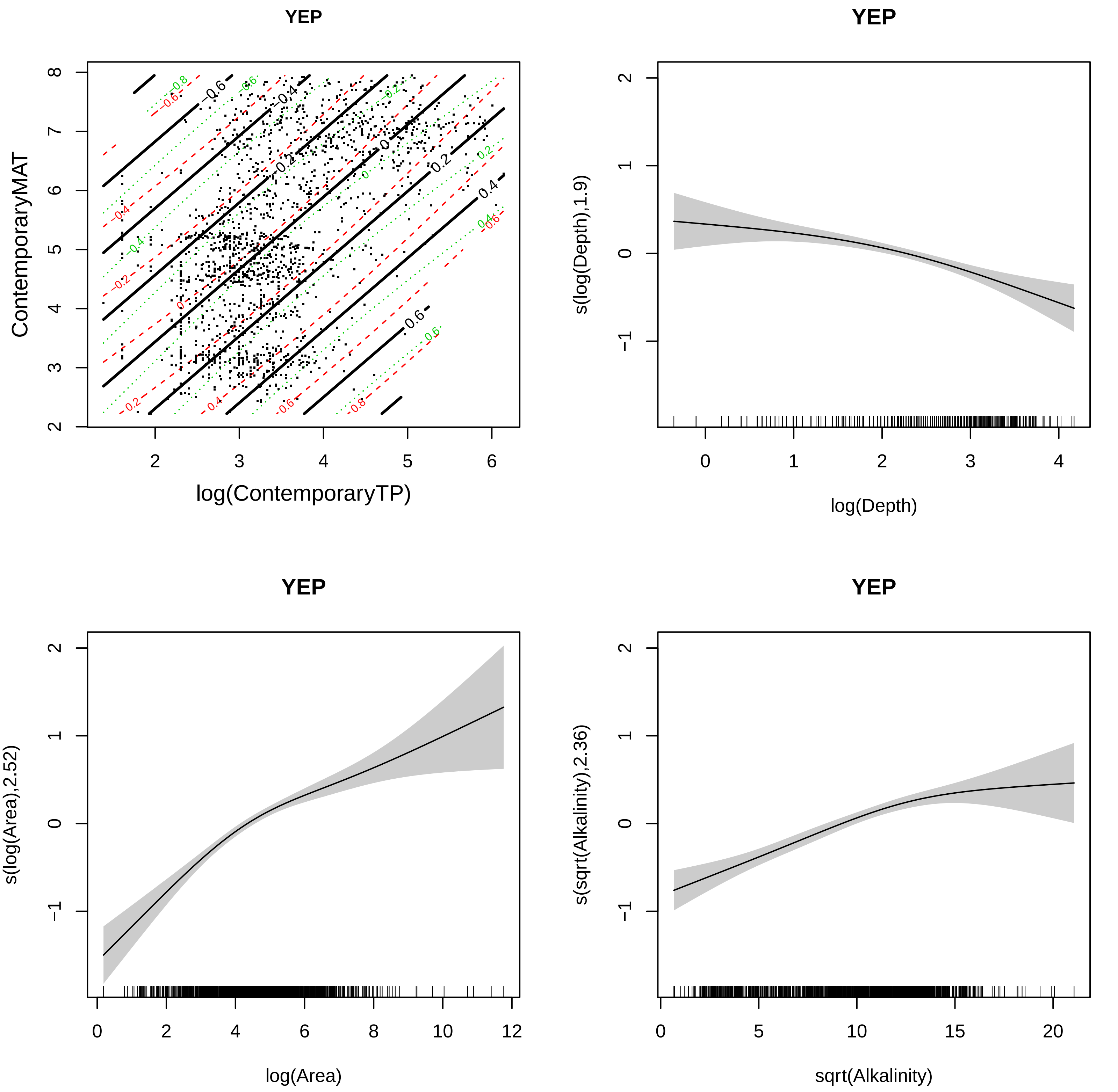
YEP
ContemporaryMAT
log(ContemporaryTP)
2 3 4 5 6 7 8
2 3 4 5 6
YEP
s(log(Depth),1.9)
log(Depth)
−1 0 1 2
0 1 2 3 4
YEP
s(log(Area),2.52)
log(Area)
−1 0 1 2
0 2 4 6 8 10 12
YEP
s(sqrt(Alkalinity),2.36)
sqrt(Alkalinity)
−1 0 1 2
0 5 10 15 20
